# Cell-drug conjugates: a novel drug delivery system for cancer therapy

**DOI:** 10.7150/thno.127171

**Published:** 2026-03-09

**Authors:** Xiaoli Cheng, Mengyuan He, Yuanye Zeng, Aixue Li, Lianghao Yuan, Yongwei Gu, Haiqiang Jiang, Jiyong Liu

**Affiliations:** 1 School of Pharmacy, Shandong University of Traditional Chinese Medicine, Jinan, 250355, China.; 2 Department of Pharmacy, Fudan University Shanghai Cancer Center; Department of Oncology, Shanghai Medical College, Fudan University, Shanghai, 200032, China.; 3 College of Pharmacy, Shanghai University of Traditional Chinese Medicine, Shanghai, 201203, China.; 4 Department of Pharmacy, Huashan Hospital, Fudan University, Shanghai, 200040, China.; 5 State Key Laboratory of Neurology and Oncology Drug Development, Nanjing, 210000, China.

**Keywords:** cell-drug conjugates, clinical translation, cancer therapy, precise targeting, drug delivery

## Abstract

Cell-drug conjugates (CDCs) represent an advanced drug delivery platform that combines living cells with therapeutics, leveraging the biological properties of cells to achieve precise targeting. By conjugating drugs onto cell surfaces, CDCs preserve cell viability and achieve functional collaborative therapy between the therapeutics and living cells. Furthermore, the pharmacokinetics of drugs can be improved by leveraging many inherent functions of cells. This review systematically introduces the construction of CDCs as drug delivery systems, including carrier cells, coupling components, and conjugation strategies*.* The advantages of CDCs as drug delivery systems and the applications of CDCs in the treatment of cancers are highlighted in this paper. Additionally, clinical trials involving CDCs for cancer therapy are analyzed. Finally, the challenges and future directions of CDCs are discussed, with the expectation that their functions will be further explored. It is hoped that this discussion will inspire researchers in their design efforts and ultimately facilitate clinical translation.

## Introduction

Cancer remains one of the leading causes of death, imposing a substantial burden on human health 1. Traditional cancer treatments, including chemotherapy, radiation, surgery, and targeted therapy, have limitations such as non-specific targets, substantial side effects, and the risk of tumor recurrence 2,3. Nanotechnology has offered promising solutions by improving drug delivery through passive accumulation via the enhanced permeability and retention (EPR) effect. However, nanoparticles (NPs) are rapidly cleared by the reticuloendothelial system (RES), limiting their efficacy 4. A biofilm-mediated biomimetic nano-drug delivery system (BNDDS) has emerged. This platform uses bio-nanotechnology to encapsulate synthetic NPs within biomimetic membranes, integrating the low immunogenicity, low toxicity, high tumor targeting, and good biocompatibility of the biofilm with the adjustability and versatility of the nanocarrier, showing promising applications in the field of precision tumor therapy 5,6. However, being fundamentally NPs, BNDDS cannot overcome the inherent limitations of their structure. A primary challenge is the interfacial energy difference between the biomimetic membrane coating and the synthetic core, which results in weak membrane-core adhesion. This makes the coating susceptible to detachment under hemodynamic shear stress, protein corona formation, or uptake by the RES, leading to particle exposure and rapid clearance 7. Furthermore, the highly heterogeneous nature of the tumor microenvironment (TME) limits the deep penetration and uniform distribution of BNDDS, resulting in suboptimal infiltration efficiency within solid tumors 8. Additionally, functional proteins on the biofilm can denature and lose activity during isolation, freeze-thawing, and extrusion preparation. These drawbacks contribute to a discrepancy where BNDDS markedly reduced effectiveness *in vivo* during clinical translation. When using an intracellular loading strategy to construct delivery systems, there is a risk of intracellular degradation towards loaded drug, which may cause damage and reduce the physiological function of the transporting cells 9. Consequently, innovative strategies are urgently required to overcome these challenges.

Cell-drug conjugates (CDCs) employ a “living carrier” strategy by using live cells as delivery vehicles. This approach fundamentally circumvents the structural bottlenecks associated with BNDDS. Therapeutics can be directly anchored to living cell membranes via covalent modification or non-covalent modification, and genetic engineering techniques can modify cells to express therapeutic proteins on their surface or synthesize them *in situ*, thereby achieving functional integration and collaborative therapy between the therapeutics and living cells. The circulation persistence of CDCs is determined by the metabolic properties of their cellular carriers. For instance, red blood cell-based CDCs capitalize on the cells' natural 120-day lifespan to achieve “ultra-long sustained release” 10. CDCs preserve native cellular functions. Immune cells intrinsically possess chemotaxis, deformability, and transendothelial migration of monocytes, enabling active penetration of tumor stroma along chemokine gradients. As demonstrated, neutrophil-based CDCs sustain migration velocities >30 µm/min even in abnormally high-pressure zones, thereby delivering drugs directly to hypoxic cores and achieving significantly enhanced tumor penetration depth 11,12. Moreover, T cell-based CDCs deliver Interleukin-15 (IL-15) while simultaneously recognizing tumor antigens through T cell receptor (TCR) engagement, enabling dual therapeutic-immunological targeting 13. Similarly, platelet-based CDCs use P-selectin to capture circulating tumor cells (CTCs), achieving synchronous drug deployment and target elimination of tumor cells 14. Therefore, relative to BNDDS, CDCs integrate cellular physiological capacities with therapeutic efficacy to represent a more promising platform for cancer therapies. This paper reviews the construction of CDCs as drug delivery systems, including carrier cells, coupling components, and conjugation strategies. In addition, this paper emphasizes the advantages of CDCs as drug delivery systems and the diverse range of therapeutic approaches of CDCs used for cancer treatment, including chemotherapy, immunotherapy, gene therapy, and combination therapy. The current clinical trials of CDCs are analyzed, providing insights into their translational progress and existing limitations. Finally, we discuss emerging opportunities and key challenges in the future development of CDCs, aiming to offer perspectives that may guide their rational design and clinical translation.

## Construction of CDCs as Drug Delivery Systems

### Live Cells as Delivery Vehicles

**Figure 1:**
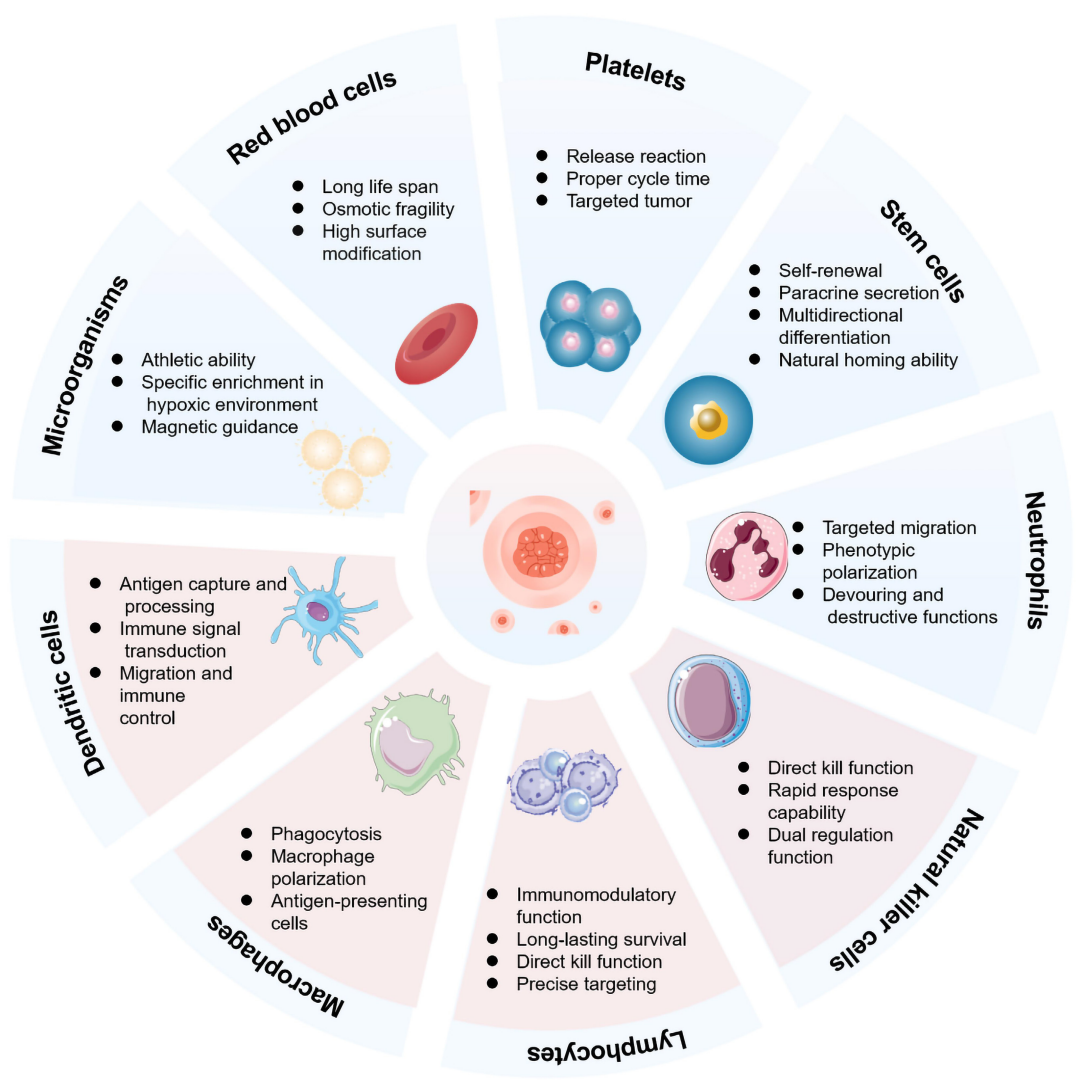
Functional advantages of different cell types. The advantages of using red blood cells, platelets, stem cells, neutrophils, natural killer cells, lymphocytes, macrophages, dendritic cells, and microorganisms as living carriers in cell-drug conjugates (CDCs).

Due to their inherent biological properties, different types of carrier cells exhibit distinct functional advantages for drug conjugates **(Figure 1).** This section systematically analyzes the functional attributes of erythrocytes, platelets, stem cells, immune cells, and microorganisms in various cancer treatment contexts. A comprehensive analysis is conducted across different cell types for CDCs in** Table 1**. The aim is to establish a scientific rationale for selecting the optimal cell carrier for a given application.

#### Red Blood Cells

The expression of Cluster of Differentiation 47 (CD47) glycoprotein on membranes of red blood cells (RBCs) transmits an inhibitory “don't eat me” signal via the signal regulatory protein alpha (SIRPα) pathway, which prevents macrophage uptake and prolongs the time of RBCs *in vivo* without affecting RBC lifespan 15. Ji *et al*. utilized Staphylococcal protein A (SPA) as a linker to immobilize antibodies on the surfaces of RBCs. They used anti-tumor necrosis factor alpha (anti-TNFα) as a model and demonstrated the stability and functionality of these modified RBCs 16. RBCs serve as inherent long-circulation vehicles that enable sustained drug release through surface adsorption. For instance, conjugates of small-molecule anticancer drugs with the polyzwitterion poly(2-(N-oxide-N,N-diethylamino)ethyl methacrylate) (OPDEA) can dynamically adsorb onto RBC surfaces. This adsorption promotes drug extravasation into tumor tissue through a process known as adsorption-mediated transcytosis (AMT) 17. In a lung metastasis model of melanoma, RBC-coupled nanoparticles loaded with doxorubicin (DOX) demonstrated a 120-fold increase in drug accumulation within metastatic foci compared to the free drug, while also maintaining stability under high shear stress 18. However, RBCs lack active chemotactic ability and cannot deeply penetrate solid tumor tissue through passive diffusion alone 19. Consequently, this approach is better suited for treating hematogenously disseminated metastases or hematological malignancies. Current research primarily focuses on modifying the red blood cell surface with targeting ligands to enhance their accumulation and penetration into solid tumors.

#### Platelets

**Figure 2:**
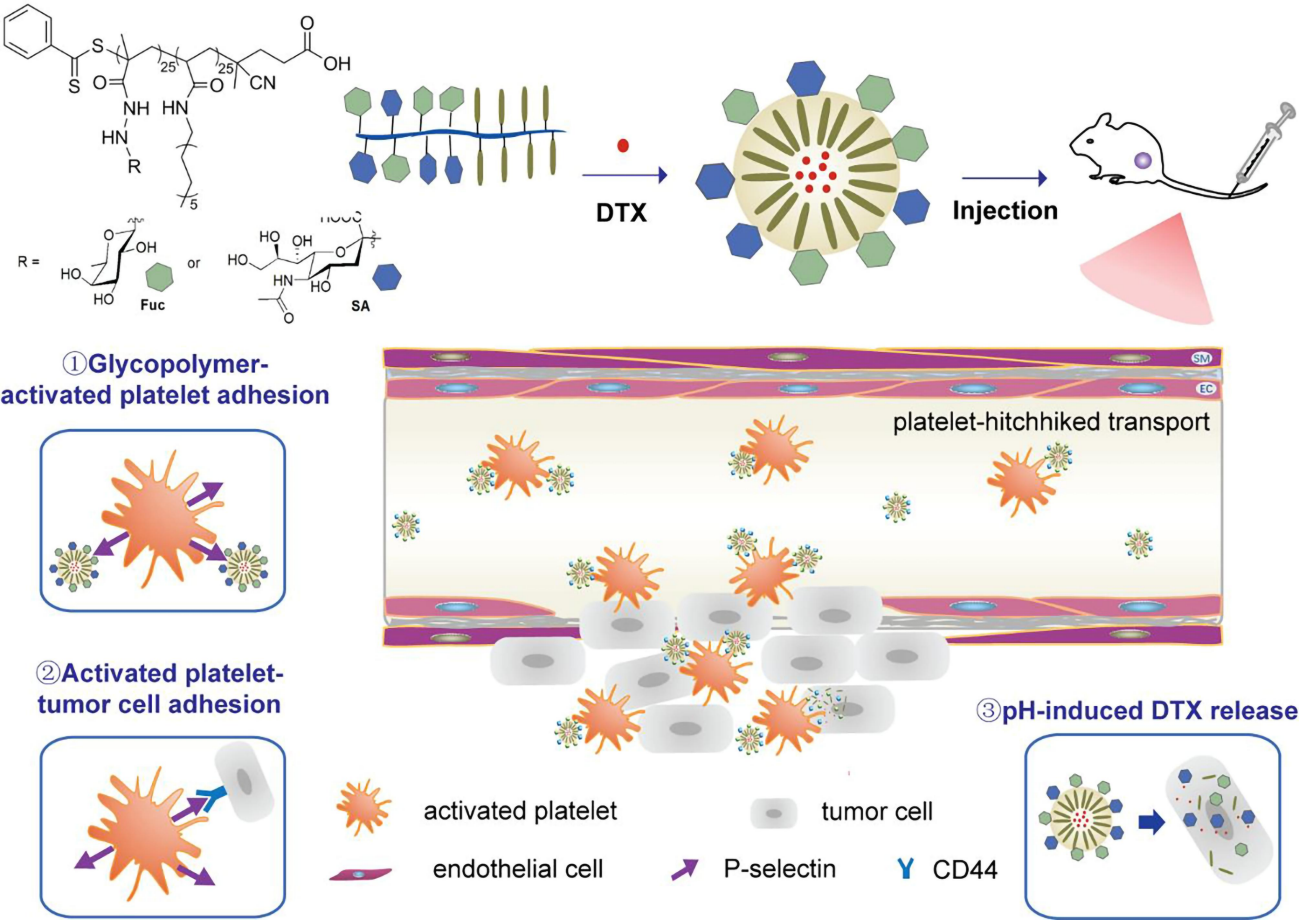
Schematic illustration of the preparation of docetaxel (DTX) loaded (red dots)-fucose (Fuc, green), sialic acid (SA, blue), and dodecylamine (khaki) amphiphilic glycopolymeric NPs and their platelet-mediated tumor-targeting and anti-tumor metastasis properties. Adapted with permission from 21. Copyright 2024, American Chemical Society.

Platelets naturally accumulate at disease sites due to their pathological homing tendency. Upon entering a diseased area, they become activated by local cues such as injured endothelium, exposed collagen, and specific signaling molecules 20. Once activated, platelets express P-selectin, a surface protein that binds to ligands like P-selectin Glycoprotein Ligand-1 (PSGL-1) and CD44 on tumor cells. Zhang *et al*. designed amphiphilic glycopolymer micellar NPs, which mimic PSGL-1, that specifically bind to activated platelets. The directed tumor-targeting effect of activated platelets has significantly improved the tumor accumulation capacity of the glycopolymeric NPs, with up to 21.0% found in tumors within the initial 0.2 h **(Figure 2)** 21. In addition, platelets have been engineered to deliver immune checkpoint inhibitors with high efficiency, enabling precise and effective tumor therapy. In a triple-negative breast cancer model, anti-PDL1 antibodies (engineered monoclonal antibodies against programmed-death ligand 1) were covalently conjugated to platelet surfaces. Upon activation at the surgical site, the platelet-based CDCs not only released the antibodies to block immunosuppressive signals but also generated platelet-derived microparticles that further expanded the drug distribution. This combined action effectively suppressed tumor recurrence and metastasis 22. Although platelets have a shorter circulatory lifespan compared to RBCs, they exhibit a high affinity for CTCs and a unique ability to accumulate in the pre-metastatic niche. These characteristics confer a distinct value to platelets in controlling post-surgical cancer metastasis.

#### Stem Cells

Stem cells, particularly mesenchymal stem cells (MSCs), possess a natural tumor-homing tendency and immunomodulatory properties 23. In models of deep-seated and poorly vascularized solid tumors, MSCs can migrate toward the tumor site by following gradients of tumor-secreted chemokines 24. For instance, one study demonstrated that MSCs conjugated with paclitaxel (PTX)-carrying NPs could significantly inhibit tumor growth and prolong survival in a mouse model of ovarian cancer 25. In addition, various therapeutic genes, including immunomodulatory genes, proapoptotic genes, suicide genes, and antiangiogenic factor-encoding genes, can be loaded into MSCs via either viral-based gene transduction or non-viral gene transfection 24. The core therapeutic mechanism of stem cells is attributed primarily to their potent paracrine activity. Paracrine signaling dampens inflammation and prevents cell death while concurrently promoting the growth of new blood vessels and the proliferation of stem cells 26. The innate ability of stem cells to self-renew and survive in the TME equips them for long-acting drug delivery. However, the pleiotropic biological activities of MSCs, including their interactions with tumor-associated stromal and immune components, necessitate careful engineering and safety evaluation when applied as carriers of CDCs.

#### Immune Cells

**Figure 3:**
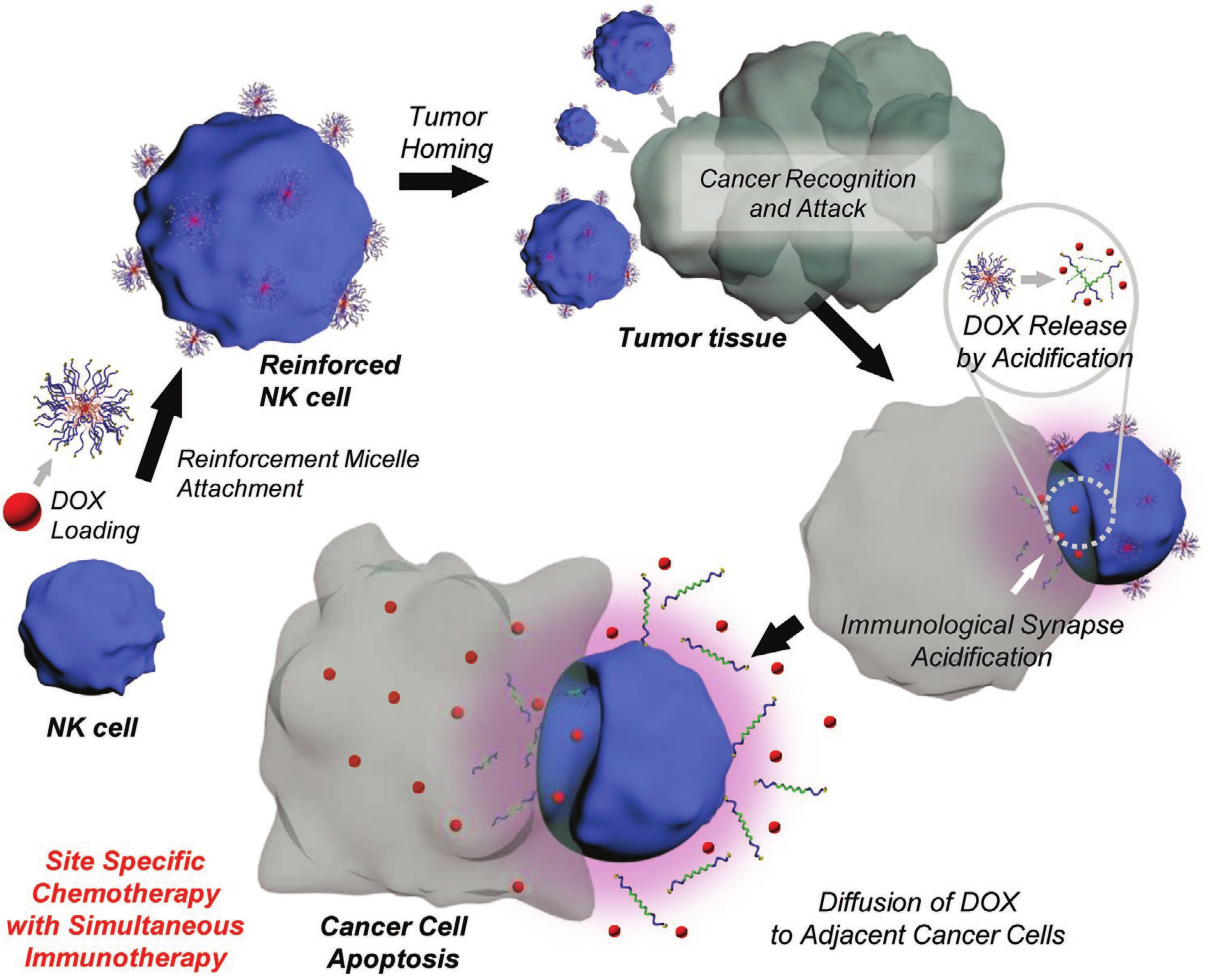
Schematic illustration of the reinforced natural killer cell (ReNK) system and its anti-cancer effect upon encountering cancer cells. Adapted with permission from 34. Copyright 2020, Wiley-VCH Verlag GmbH & Co. KGaA, Weinheim.

T lymphocyte (T cell) activation is a process that requires two signals from antigen-presenting cells: antigen recognition and co-stimulation 27. Activated T cells, specifically cytotoxic T cells, can precisely target and destroy tumor cells 28. In MicroSatellite Stable (MSS) colorectal and pancreatic cancers, which require profound immune activation, T cell-based CDCs can precisely deliver immunomodulators to tumor-infiltrating lymphocytes (TILs), overcoming the barrier of “cold tumors” 29,30. Tang *et al*. developed a T cell-based CDC that is activated by TCR signaling 31. The system binds “backpacks” of immunostimulatory drugs on the surface of T cells. Upon recognizing their specific antigen, the T cells release the payload locally within the tumor, precisely reactivating TILs, avoiding systemic toxicity, and significantly enhancing anti-tumor efficacy. However, the therapeutic use of T cells is constrained by their short circulating lifespan and limited availability from patients with lymphopenia following intensive radiotherapy or chemotherapy. In such cases, NK cells present a viable alternative. Unlike T cells, Natural Killer (NK) cells can identify and eliminate tumor cells without prior sensitization and do not require Human Leukocyte Antigen (HLA) matching 32,33. Kim *et al.* engineered NK cell-based CDCs by anchoring drug-loaded, acid-responsive nanoparticles (ReMi) onto NK cell surfaces via maleimide-thiol conjugation chemistry. The strategy enhanced tumor cell elimination, achieving significantly improved therapeutic efficacy compared to standalone therapies (**Figure 3**) 34. However, the therapeutic potential of NK cells is limited by their low abundance in peripheral blood and their frequent dormant or inactive state 35. To overcome these limitations, cationic nanoparticle (cNP)-based activation strategies 36 and magnetic nanoparticle-targeted delivery platforms 37 have been successively developed. It should be noted that NK cells demonstrate limited penetration into solid tumors compared to T cells, necessitating the use of adjuncts like IL-15 superagonists or CAR-NK technology to sustain their viability and functional activity 38,39.

As first responders of innate immunity, circulating neutrophils (NEs) and tissue-resident macrophages rapidly sense inflammatory cues in the TME and are recruited to diseased tissues via chemokine gradients and hypoxia-associated signals 40-42. Although both types of cells have some tumor-homing ability, NEs are excellent for targeting acute inflammation, and macrophages are key players in chronic inflammation 43,44. Leveraging macrophage tumor tropism, M1 macrophage DOX nanoparticle conjugates (MA-DMPM) achieved efficient tumor targeting and induced apoptosis in 63.33% of tumor cells 45. Chen *et al*. further covalently anchored β-elemene-loaded germanium sulfide nanosonosensitizers (GeSNSs@ELE) onto macrophage surfaces through maleimide-thiol chemistry. Macrophage hitchhiking delivery of β-elemene-loaded GeSNSs not only achieves high accumulation in tumor regions and suppresses tumor growth under ultrasound treatment but also effectively remodels the immunosuppressive tumor environment, thereby facilitating enhanced sonodynamic chemoimmunotherapy 46. Macrophage-based strategy is applicable in scenarios such as sustained immune remodeling, hypoxia targeting, or blocking pre-metastatic niches. However, vigilance against potential M2 phenotype-mediated tumor progression and complement activation is necessary 47,48. The ability of NEs to rapidly migrate to inflammatory sites via L-selectin/PSGL-1 mediation makes them well-suited for delivering drugs across the blood-brain barrier (BBB). A singlet oxygen (^1^O2)-cleavable nanocarrier, loaded with a semiconducting polymer, programmed death-ligand 1 (PD-L1) siRNA, and iron oxide (Fe_3_O_4_) NPs, was conjugated to neutrophils, enabling efficient BBB penetration and targeted delivery to glioblastoma (GBM), resulting in significant tumor suppression and prolonged survival in murine models 49. Despite the outstanding advantage of NEs to cross the BBB and infiltrate necrotic tumor cores, the clinical translation of neutrophil-based therapies is significantly limited by their short half-life *in vivo* and functional heterogeneity. Consequently, rigorous and dynamic assessment of donor neutrophil quantity and activity is essential before treatment.

Dendritic cells (DCs) activate T-cell responses through antigen presentation while modulating autoimmunity 50. DC-based CDCs are designed to function through local “*in situ* activation” rather than by achieving deep tissue penetration. For instance, Wang *et al*. enhanced this effect by modifying DC membranes with azide groups via metabolic glycoengineering, followed by a copper-free click conjugation of IL-15. This approach boosted T-cell responses and strengthened anti-tumor immunity 51. A critical consideration when selecting DCs as a therapeutic vehicle, however, is balancing their maturation and immunogenic potential against the risk of inducing autoimmune disease through excessive immune activation.

#### Microorganisms

**Figure 4:**
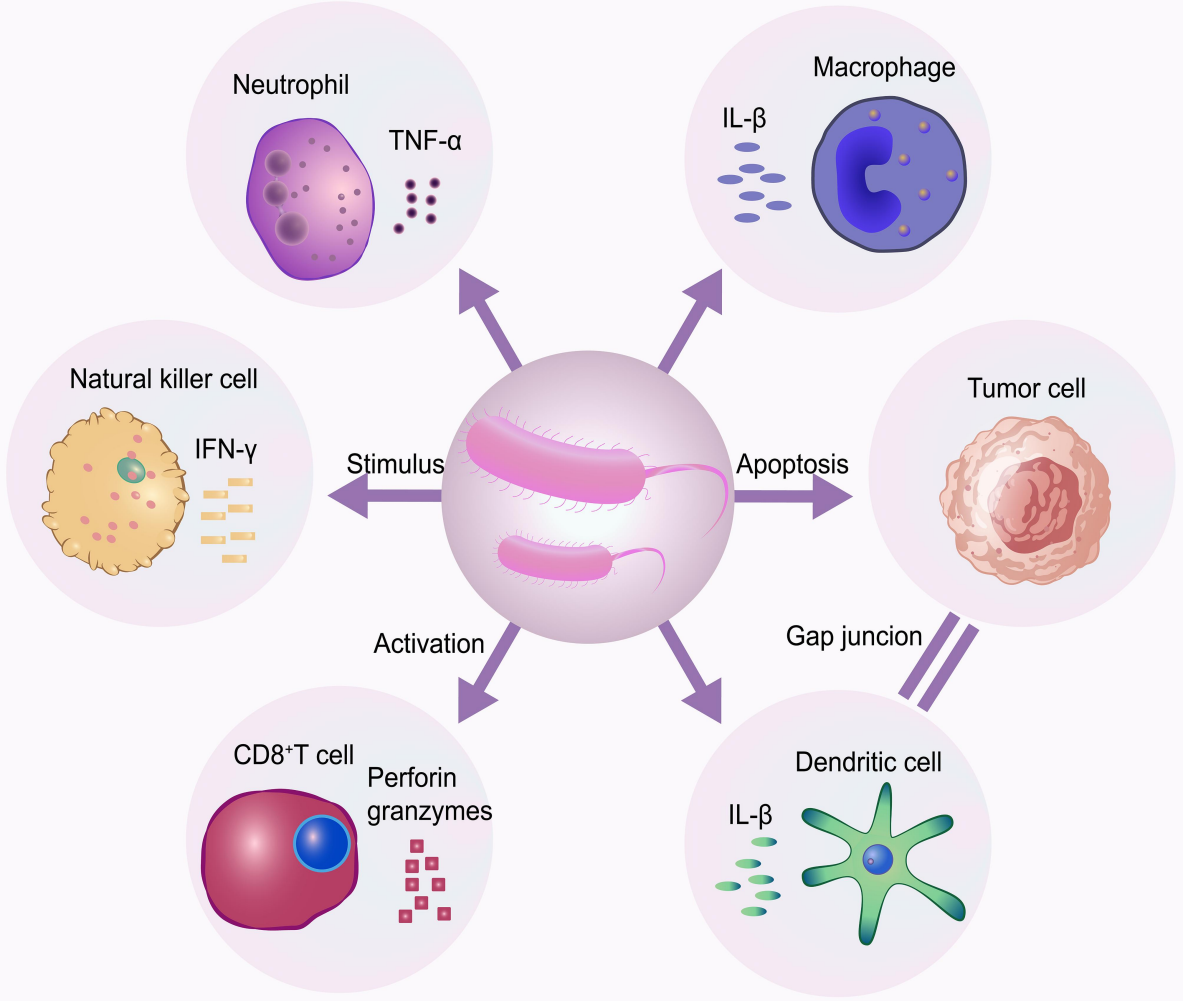
Mechanisms by which bacteria target tumors. Tumor-targeting bacteria preferentially colonize hypoxic tumor regions and elicit antitumor effects through immune activation and direct tumor cell killing. CD8+ T cell, cytotoxic T lymphocyte; TNF-α, tumor necrosis factor alpha; IL-β, interleukin-1beta; IFN-γ, interferon gamma.

The antitumor efficacy of bacteria is partly attributable to their immunomodulatory properties. The inflammatory response triggered by a bacterial infection can indirectly eliminate tumor cells by activating antitumor immunity 52. Furthermore, bacteria possess the ability to selectively colonize and proliferate within the hypoxic regions of tumors. This innate targeting specificity, when enhanced through engineering to allow for magnetic guidance, enables them to reach deep-seated tumors or cross the blood-brain barrier. Bacterial overgrowth in tumors induces tumor regression via several different mechanisms **(Figure 4)**. In a study by Felfoul *et al*., magnetotactic bacteria MC-1, under external magnetic guidance, successfully delivered 7-ethyl-10-hydroxycamptothecin (SN-38)-loaded liposomes to the target site 53. The engineered bacteria are particularly suited for targeting the core of “hard tumors,” which are often impermeable, avascular, and immunosuppressive 54. However, to mitigate risks such as endotoxin release, systemic infection, and the horizontal transfer of antibiotic resistance genes, robust safety measures are required. These include the use of auxotrophic strains, LPS-attenuated variants, and inducible suicide switches.

### Representative Coupling Components

#### Janus NPs

NPs lack intrinsic targeting capability and can only bind to live cells under* ex vivo*. To enable autonomous* in vivo* targeting of specific cells, Janus NPs with dual functionalities were engineered. By asymmetrically functionalizing two distinct hemispheres of the NPs with complementary modifications, the NPs achieve both cellular targeting and stable surface adhesion without rapid internalization 55. For instance, Zhang *et al*. designed a nanosystem composed of Janus Au/mesoporous silica core/shell nanoparticles (Janus NPs). The Janus NPs are composed of an Au core and a pirfenidone-loaded mesoporous silica shell carrying two different targeting moieties, reactive oxygen species (ROS)-stimulative thioketal grafted methoxy poly (ethylene glycol) (mPEG-TK) and 1,2-distearoyl-sn-glycero-3-phosphoethanolamine (DSPE). The design aims to anchor the Janus NPs on the cell membranes of MSCs via DSPE modification on the hemispherical surfaces and inhibit their cellular endocytosis by the passivation of PEG modification on the other hemisphere of the particle surfaces. By creating such dual-functionalized Janus NPs, ROS-responsive release of pirfenidone in the lung is achieved to provide a favorable microenvironment for the injected MSCs, thereby enhancing the treatment outcome of MSCs in lung cancer 56. Janus NPs, as particles with anisotropic surface chemistry, can modify one face with a cell-targeting ligand and the other face with a ligand, thus binding to effector cells to trigger target cell-killing capability. Liu *et al*. developed an entirely synthetic, multivalent, Janus nanotherapeutic platform, called synthetic nanoparticle antibodies (SNAbs). SNAbs, with phage-display-identified cell-targeting ligands on one “face” and Fc-mimicking ligands on the opposite face, were synthesized using a custom, multistep, solid-phase chemistry method. SNAbs efficiently targeted and depleted myeloid-derived immune-suppressor cells (MDSCs) from mouse-tumor and rat-trauma models,* ex vivo*, enabling enhanced T cell and NK cell infiltration into tumors 57.

#### Liposome-Based NPs

Due to their phospholipid bilayer architecture, liposomes enable the efficient encapsulation of both hydrophilic and hydrophobic drugs, while offering a highly adaptable surface for chemical and biological functionalization 58. In the design of CDCs, liposomes can be engineered to mediate receptor-dependent coupling to cells. For instance, polysialic acid (PSA), a hydrophilic and non-immunogenic biopolymer, has been employed to functionalize pixantrone-loaded liposomes through PSA-octadecyl carboxylate conjugation. By exploiting PSA receptors expressed on peripheral blood neutrophils, these liposomes achieved efficient binding to neutrophils and enhanced antitumor efficacy *in vivo* 59. Similarly, liposomes modified with anti-lymphocyte antigen 6 complex, locus G(anti-Ly6G) antibodies could specifically target and combine with NEs. These engineered liposomes were subsequently transported to post-surgical inflammatory tumor sites, enabling co-delivery of DOX and the stimulator of interferon genes (STING) 60. Such Immune cell-liposome conjugates bound through antibody-mediated recognition to achieve spatially controlled drug delivery via inflammation-responsive cellular carriers.

Polyethylene glycol (PEG) modification has been widely applied to prolong circulation time and reduce nonspecific interactions of liposomes 61. Reactive functional groups, such as maleimide moieties introduced at PEG termini, enable site-specific covalent conjugation with thiol groups on cell-surface proteins, thereby stabilizing cell-liposome assemblies 62. Lipid-polymer hybrid nanoparticles further expand this design space, as they can self-assemble into functionalized carriers with surface-exposed maleimide groups that allow direct and selective binding to macrophages without additional modification steps 63. More recently, multifunctional liposome-based systems have been developed. Peptide-liposome hybrid nanoparticles represent a notable example of this trend. In a reported study, DOX·HCl was encapsulated in the matrix metalloproteinase 2 (MMP-2) responsive peptide-liposome (D@ML), followed by surface modification with lipoteichoic acid to enable monocyte binding via CD14 receptors. Upon binding to monocytes, the engineered liposomes are carried to the tumor core. DOX·HCl was then released by the MMP-2 response, inducing immunogenic cell death. 64. Such designs illustrate that liposomes can serve as programmable coupling modules of CDCs, coordinating tumor microenvironment-triggered drug release with immune cell-mediated transport.

#### Immunotherapeutic Agents

PD-L1 is extensively expressed in tumor cells and certain immune cells, where it binds to PD-1 on T cells, hence impeding the immunological recognition capabilities of T cells 65. The first PDL1 inhibitor, atezolizumab, has recently been granted accelerated approval by the US Food and Drug Administration (FDA) 66. Despite remarkable progress, the current immune checkpoint blockade (ICB)-based treatments still have many limitations 67. Side effects, such as autoimmune disorders, have often occurred in those undergoing ICB therapy. To address these limitations, researchers have begun exploring live cell-based conjugation strategies to achieve the spatiotemporally precise delivery of immune checkpoint inhibitors. This approach is further enabled by the intrinsic properties of these inhibitors: most are structurally stable monoclonal antibodies or their derivatives, featuring well-defined binding sites and long *in vivo* half-lives, which facilitate chemical or biological conjugation to cells. For instance, anti-PD-L1 antibodies were conjugated with the crosslinker Sulfosuccinimidyl 4-(N-maleimidomethyl) cyclohexane-1-carboxylate (Sulfo-SMCC), introducing maleimide groups that selectively bind to surface thiols (-SH) on platelets. With platelet-mediated delivery, aPD-L1 effectively targeted cancer cells post-surgery while minimizing off-target effects. Studies confirmed that aPD-L1 binds stably to resting platelets but is efficiently released upon platelet activation 22. Li* et al*. further engineered multifunctional platelet-based CDCs by co-conjugating the vascular disrupting agent vadimezan with aPD-L1, achieving synergistic tumor vasculature disruption and immune activation 68. These studies collectively highlight the feasibility of integrating immunotherapeutic agents into the platforms of CDCs.

### Conjugation Strategies of CDCs

**Figure 5:**
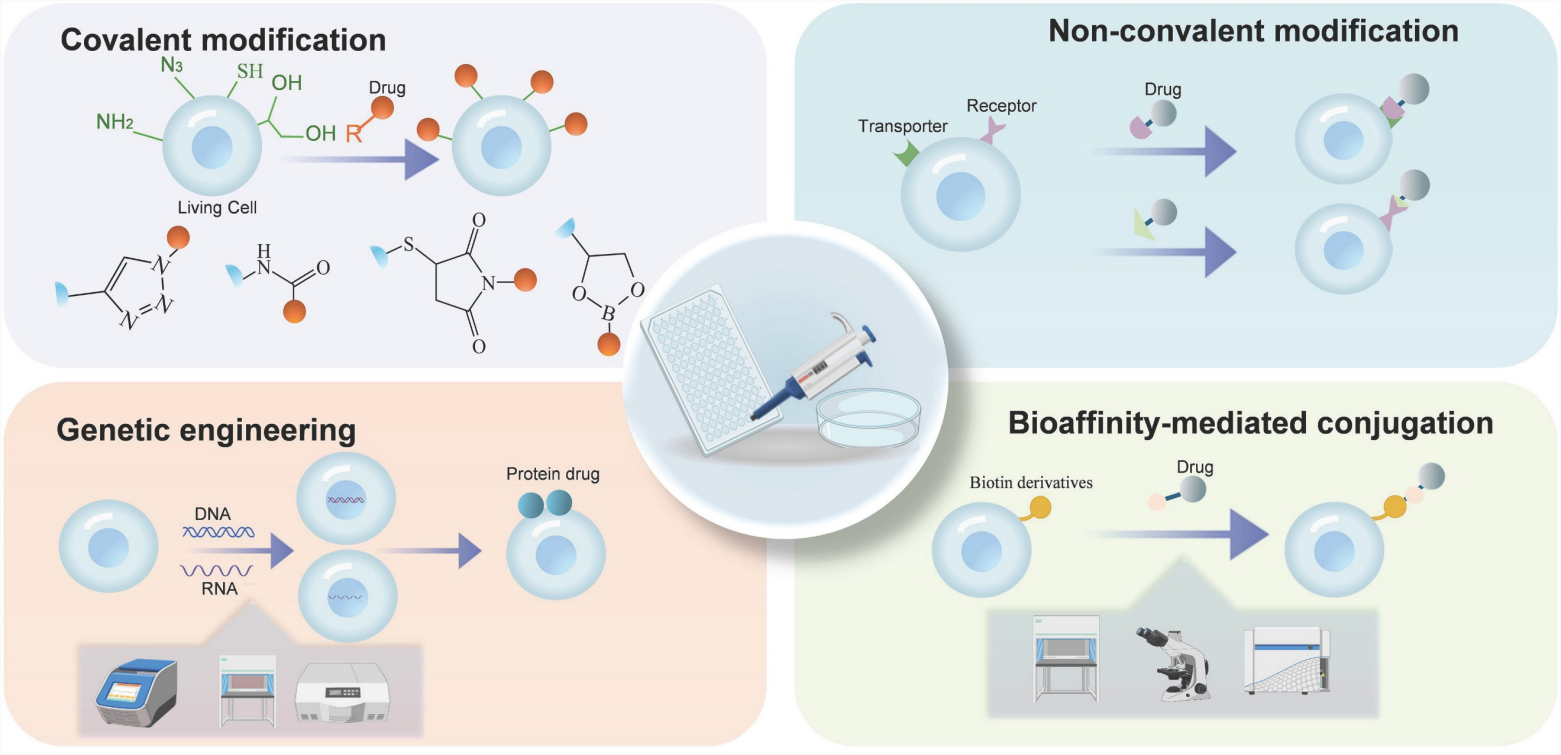
Schematics of covalent modification, non-covalent modification, bioaffinity-mediated conjugation, and genetic engineering.

The construction of CDCs is central to achieving their biological function and therapeutic efficacy. Successful assembly of CDCs requires the stable loading and controlled release of therapeutic agents on the cell surface, while maintaining cell membrane integrity and normal cellular physiology. The primary strategies for constructing CDCs include covalent modification, non-covalent modification, bioaffinity-mediated conjugation, and genetic engineering** (Figure 5)**. Each strategy offers advantages and limitations concerning conjugation stability, modification biocompatibility, and *in vivo* performance.

#### Covalent Modification

Cell surfaces are rich in proteins bearing reactive functional groups, particularly primary amines (-NH₂) and thiols (-SH), which serve as key sites for covalent conjugation. 69. Succinimidyl esters selectively react with amines, while maleimides preferentially bind thiols. 70. Using maleimide chemistry, Ayer *et al*. achieved efficient NP conjugation on T-cell surfaces (~100 NPs per cell) without inducing cytotoxicity or impairing cellular functions 71. Subsequent studies confirmed that thiol-maleimide modification did not affect T-cell proliferation, target recognition, or BBB migration, indicating its potential for medication delivery to the central nervous system (CNS). Compared to coupling methods that require additional catalysts to activate the reaction, biorthogonal click chemistry offers a more bio-friendly approach for the stable conjugation of functional or therapeutic moieties with living cells 72. Biorthogonal click chemistry combines metabolic engineering with specific bioorthogonal covalent reactions. Cells are first engineered to metabolically incorporate non-natural functional groups—such as azides or alkynes—which become selectively displayed on cell-surface glycoproteins or glycolipids. Subsequently, these bioorthogonal handles enable covalent ligation with functional or therapeutic molecules carrying complementary reactive groups via strain-promoted azide-alkyne cycloaddition (SPAAC) reactions 73,74. These reactions proceed selectively in aqueous solutions without the need for catalysts, which makes them particularly well-suited for the modification of live cell surfaces. Prior research has applied this method to successfully conjugate PTX NPs to the surface of MSCs, significantly prolonging drug retention and resulting in effective tumor suppression *in vivo* 75. Enzymatic ligation reactions can also achieve effective connections between living cells and drugs. Typically, enzymatic ligation reactions require the genetic engineering of cells to express specific recognition sequences or tag proteins on their surface. Subsequently, the corresponding enzyme catalyzes the formation of a stable covalent bond under mild conditions 76,77. Compared to biorthogonal click chemistry, enzyme-mediated conjugation offers superior precision and reproducibility, albeit with greater procedural complexity. Notably, the spatiotemporal control of enzymatic ligation can be further enhanced through the use of photoactivatable enzyme variants, enabling precise control of drug release.

Although covalent modifications provide high stability, they suffer from nonspecific binding—reactive groups may disrupt membrane protein function. For instance, N-hydroxysuccinimide ester (NHS) can react not only with lysine residues but also with cysteine and other residues, potentially interfering with signaling activity and other biological processes 78,79. These off-target effects can be mitigated by optimizing reaction stoichiometry or introducing affinity-selective peptides 80.

#### Non-Covalent Modification

**Figure 6:**
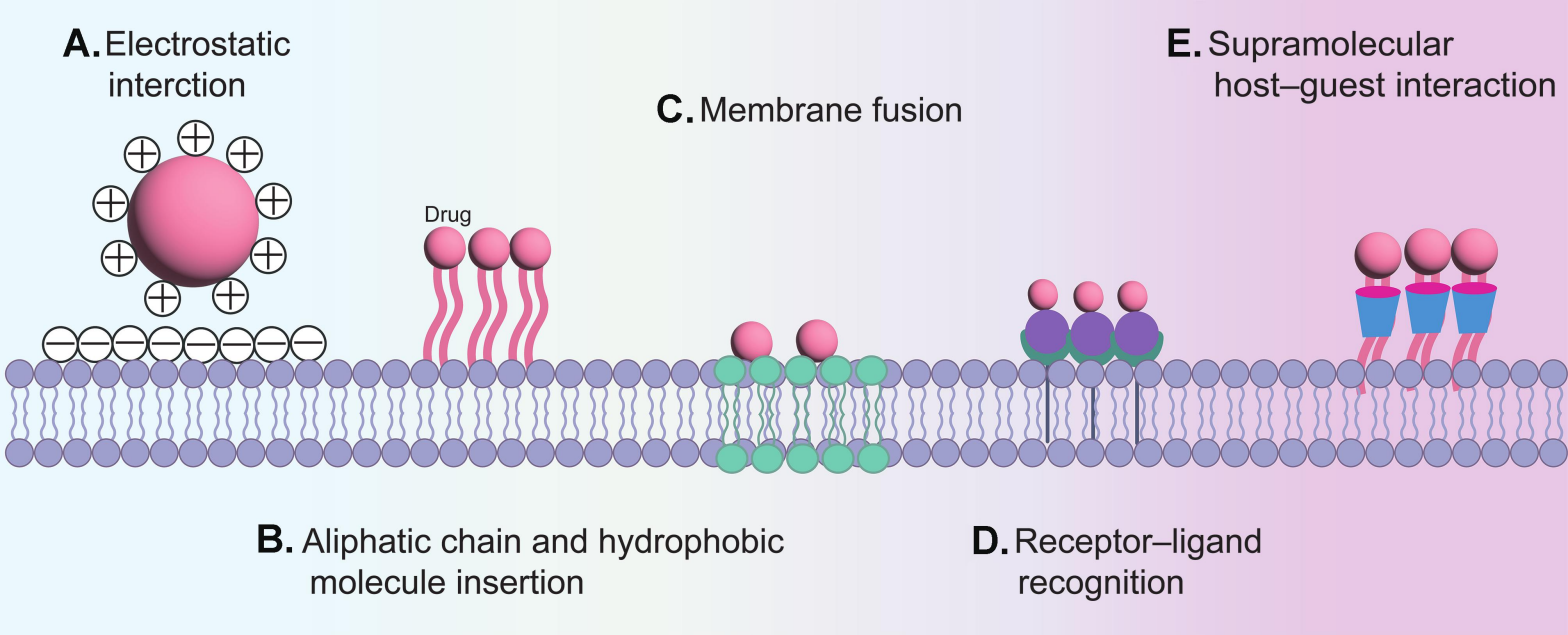
Non-covalent modification. (A) Electrostatic interaction. (B) Aliphatic chain and hydrophobic molecule insertion. (C) Membrane fusion. (D) Receptor-ligand recognition. (E) Supramolecular host-guest interaction.

Non-covalent modification is a key strategy in the preparation of CDCs, offering greater flexibility and controllability compared to covalent modification. This approach leverages physicochemical and biological interactions, including electrostatic forces, hydrophobic effects, membrane fusion, specific recognition, and host-guest binding, to conjugate therapeutic agents with target cells. **(Figure 6)**.

Hydrophobic interactions are essential to the architecture of the lipid bilayer in cell membranes, and lipid molecules can be efficiently integrated into the membrane by hydrophobic insertion methods 81. Studies demonstrate that elongated saturated lipid chains enhance membrane anchoring stability, which is critical for polymer integration within phospholipid bilayers 82. DNA-lipid conjugates leverage this principle to achieve cellular binding 83. Its advantage lies in preserving membrane protein functionality while exhibiting gentler effects compared to covalent modifications. Moreover, the compositional similarity between liposomes and cell membranes enables direct delivery via membrane fusion. For instance, Yousaf *et al*. synthesized many large vesicles containing oxyamine or ketone functional groups. Subsequently, oxyamines and ketones were presented on the cell surface due to the membrane fusion of liposomes containing ketone and oxyamine groups with fibroblasts 84. The sialic acid residues of glycoproteins confer intrinsic negative charges to plasma membranes 85. Cationic materials, including poly (styrene sulfonate) (PSS), poly (acrylic acid) (PAA), and poly-diallyldimethyl ammonium chloride, together with cationic NPs, are electrostatically attracted to cell surfaces 86. For example, Ca^2+^ was suggested to function as an “adhesive” element between the DC membrane and polydopamine, due to the electrostatic contact between Ca^2+^ and both the cell membrane and polydopamine. 87. Besides these nonspecific interactions, NPs coated with hyaluronic acid (HA) were reported to bind to cell surfaces via receptor-mediated recognition 88. Conjugating drugs to cells also can be achieved by functionalizing the surfaces of NPs with monoclonal antibodies that target specific cell surface antigens, such as CD45 or CD11b 89.

Host-guest interactions represent a supramolecular modification strategy 90. Owing to its hydrophobic cavity, β-cyclodextrin can reversibly bind to adamantane (binding affinity: 10⁴-10⁵ M⁻¹), providing a foundation for constructing dynamic coupling systems 91. Moreover, natural polyphenols contain abundant catechol or galloyl groups in their structure, enabling multivalent interactions with various substrates 92. The unique structural and chemical properties of polyphenols have facilitated the development of a series of polyphenol-functionalized nanostructures (PNAs), including nanocomposites, NPs, and nano-coatings 93. Notably, the catechol or galloyl groups on the surface of PNAs confer cell-adhesive capabilities to functionalized substrates, allowing them to bind to various cell types.

#### Bioaffinity-Mediated Conjugation

The noncovalent interaction between biotin and avidin/streptavidin is among the strongest known protein-ligand interactions and is therefore often regarded as a “quasi-covalent” linkage in biomedical applications 94. Biotin derivatives can be covalently anchored to the cell surface through reactions with amino or thiol groups on membrane proteins; subsequently, leveraging the high-affinity recognition of biotin by avidin or streptavidin, these proteins act as molecular bridges to mediate the stable assembly of biotinylated drugs on the cell surface 95. A representative example is the erythrocyte-tissue plasminogen activator (tPA) system assembled via streptavidin-biotin coupling, in which biotinylated erythrocyte membranes anchor tPA through streptavidin bridging. This strategy enables each erythrocyte to carry approximately 10⁴-10⁵ tPA molecules without compromising cellular integrity or circulation lifespan 96. Beyond directly conjugating proteins to cells, bioaffinity-mediated conjugation has also been extended to the coupling between cells and nanoparticles. Ye Feng *et al*. developed a biotinylated erythrocyte-poly(lactic-co-glycolic acid) (PLGA) nanoparticle hybrid platform (bE-NPs) using avidin-biotin interactions, in which RBCs were modified with NHS-biotin and PEG-PLGA nanoparticles were functionalized with streptavidin. bE-NPs exhibited significantly enhanced tumor accumulation compared with unmodified nanoparticles, highlighting the synergistic benefits of integrating cellular carriers with nanotherapeutics through bioaffinity interactions 97.

Despite its advantages, bioaffinity-mediated conjugation has its limitations. The immunogenicity of avidin/streptavidin, potential crosslinking-induced membrane perturbation, and limited controllability over ligand density remain critical challenges for clinical translation. Future efforts should prioritize reducing immunogenicity and improving spatial controllability by replacing avidin/streptavidin with low-immunogenic or protein-free affinity modules.

#### Genetic Engineering

Genetic engineering offers a distinct approach for creating CDCs. Cells are engineered to become intrinsic drug producers by introducing genes that encode therapeutic proteins, effectively transforming them into sustained “living factories” of the therapeutics. This approach, in which cells are genetically programmed to produce therapeutic proteins, represents a more advanced form of integrated conjugation. Unlike conventional methods, the therapeutic agent is not an externally attached payload, but a functional component of the engineered cell 98. Consequently, drug conjugation is achieved through endogenous cellular biosynthesis rather than external chemical linkage. A key advantage of this strategy is the ability to achieve localized and controlled expression of the therapeutic protein on the cell membrane. To ensure the engineered protein is stably anchored, specific genetic elements, such as signal peptides and transmembrane domains, must be incorporated into its encoding sequence. For instance, the Igk signal peptide (METDTLLLWVLLLWVPGSTGD) and the transmembrane region (amino acids Ala513-Arg561) of the platelet-derived growth factorreceptor used in the pDisplay vector 99. Please note that certain biological contexts impose specific restrictions upon the direct editing of genes. For instance, employing genetic techniques to alter mature RBCs or platelets presents considerable difficulty, as these entities are anucleate or have a functional lifespan insufficient for sustained protein production. Therefore, an alternative strategy is to transfer new genes into their precursors, such as hematopoietic stem cells (HSCs), to modify the mature RBCs or platelets genetically 100.

## Advantages of CDCs as Drug Delivery Systems

### Preservation of Cellular Integrity and Function

The cell of CDCs is not merely a “delivery vehicle” for drugs; its intrinsic biological functions are a component of the therapeutic effect itself. This represents their defining distinction from BNDDS. While cell-based intracellular drug delivery systems can indeed employ living cells as delivery carriers, the internalization of therapeutic drug payloads may disrupt cellular viability, phenotype, or effector functions 101. CDCs preserve the native biological activities of the carrier cells by confining drug loading to the cell surface. Furthermore, the preservation of cell function enables synergistic enhancement between innate cellular therapy and exogenous drug action. A prime example of this approach is the CAR-T-based conjugate. By conjugating PD-1 antibodies to CAR-T cell surfaces via lipid-anchoring techniques, researchers retain T-cell-specific cytotoxicity while achieving localized delivery of immune checkpoint inhibitors. Preclinical studies indicate that this combinatorial strategy can enhance antitumor efficacy by up to 45% 102. Combining Treg cells with nanocarriers containing immunomodulatory drugs leverages the homing ability of Treg cells to direct them to tumor sites. The localized release of immunomodulatory drugs, such as Interleukin-2 (IL-2), sustains and enhances Treg function, leading to more effective control of cancer 103. Furthermore, certain CDCs can be engineered to release stimulatory factors that enhance their own proliferation, persistence, or activity, or to promote a phenotypic shift for therapeutic purposes. For instance, attaching interferon-γ-loaded multilayer disc-shaped patches to macrophages via HA-CD44 interaction sustains their pro-inflammatory phenotype, polarizing them from M2 to M1, which enhances the anti-tumor immune response 104. This shift in treatment strategy transforms the role of the carrier from a simple “delivery” into an “integrated therapy”. The resulting synergy is not merely a combined effect from delivering multiple drugs, but stems from a systemic design informed by cell physiology and pathology.

### Optimization of Barrier Penetration, Circulation, and Targeting Capabilities

CDCs leverage the inherent biological behaviors of their cellular carriers, not just the physicochemical properties of NPs, to penetrate physiological barriers. For instance, senescent NEs, which naturally upregulate C-X-C chemokine receptor type 4 (CXCR4) to return to the bone marrow for apoptosis, can be used to co-deliver drugs directly to bone marrow lesions 105. Certain engineered immune cells, such as T cells, monocytes, and macrophages, can effectively infiltrate tissues like brain tumors and lymphomas 106,107. In one study, a polymer patch containing the enzyme catalase was attached to macrophages. These cells accumulated in the inflamed brain tissue induced by lipopolysaccharide and subsequently killed tumor cells at the lesion site 108. Furthermore, by conjugating SN-38-loaded nano-capsules to T cells equipped with lymph node-homing receptors. After intravenous administration, these CDCs accumulated preferentially in tumor-draining lymph nodes, achieving a concentration over 90 times higher than that of the free drug 109. This level of targeted enrichment is typically unattainable for BNDDS.

When constructing cell-based carriers using intracellular loading strategies, there is a risk that the loaded drug may be degraded inside the cell, which can lead to carrier cell damage and impaired physiological function 110. In contrast, the stability of CDCs is derived from the strategy of conjugating the drug onto the cell surface. For instance, Lorentz *et al*. achieved a drug circulation time of several weeks by binding the clinical therapeutic enzyme *Escherichia coli* L-asparaginase to erythrocytes *in situ* antigen-specifically 111. For nanodrug delivery, DOX NPs electrostatically adsorbed onto RBC membranes exhibited 120-fold greater pulmonary accumulation and potent suppression of melanoma lung metastases 112. Whether formed through naturally existing amino groups, thiol groups, or non-natural reactive groups introduced through metabolic glycoengineering, the resulting connections exhibit outstanding stability in physiological environments. This stability ensures that the drug can be transported to the target tissue in its intact form 75. It is worth noting that specific covalent conjugation strategies, such as coupling antigenic peptides to the Kell protein on the surface of RBCs through a reaction mediated by sorting enzyme A, can construct CDCs that remain stable in circulation for over 28 days, with circulation times significantly longer than most cell-intracellular delivery-based drug systems. 113.

CDCs leverage the inherent biological properties of living cells to achieve more natural and efficient drug targeting. This approach capitalizes on the natural homing abilities of specific cell types. For instance, MSCs actively migrate to sites of tissue injury or home to the bone marrow 114. Platelets naturally accumulate at wound sites and areas of inflammation 115. Furthermore, T cells and monocytes can be recruited by tumor-specific chemokines, cytokines, and growth factors 116. DCs mature and migrate to lymph nodes after capturing antigens, where they subsequently activate T cells 117. The targeted behaviors stem from the intrinsic ability of cells to migrate and localize in response to physiological or pathological cues, enabling them to infiltrate deep into solid tumors. These targeting mechanisms of live cells ensure that therapeutic agents can be precisely delivered to primary tumors, postsurgical residual lesions, and metastatic sites. For example, hematopoietic stem cells can be genetically engineered to express PD-L1 on their surface, enabling the targeted delivery of this immunosuppressive protein to diseased pancreatic tissue 118. Crucially, this targeting process is inherently dynamic and adaptive, enabling real-time adjustments in response to changes in the TME—capabilities that BNDDS, as static entities, lack.

### Spatiotemporally Controlled On-Demand Release

In drug delivery systems, a primary goal is to achieve targeted, efficient drug release at diseased sites while minimizing off-target effects on healthy tissues. BNDDS has made notable progress in this regard by employing materials designed to respond to specific pathophysiological cues (e.g., mildly acidic pH, specific enzymes, or elevated glutathione levels), thereby mimicking “smart” behavior 119. However, these systems predominantly rely on predetermined, relatively static response mechanisms where drug release is passively initiated by microenvironmental cues. This approach may suffer from inefficient drug release due to signal crosstalk or inadequate stimulus intensity 120. More critically, the triggered responses are typically irreversible and system-wide, posing fundamental limitations for achieving precise and efficient drug delivery in highly heterogeneous solid TME. CDCs offer superior control over drug release kinetics. Specifically, CDCs achieve on-demand drug release by precisely coupling drug liberation with the distinctive functional responses of cellular carriers in pathological microenvironments. For instance, interleukin-15 superagonist (IL-15SA)-loaded nanogels utilize both electrostatic interactions and CD45 antibody-mediated specific binding to T cells 31. This system achieves precise drug delivery, with primary IL-15SA release occurring at immune synapses formed between T cells and tumor cells, fulfilling both “on-demand” and “on-site” therapeutic requirements. Moreover, CDCs capitalize on the innate homing capabilities of cells to establish natural “targeting coordinates” for site-specific drug delivery. A representative example involves platelet-bound cytotoxic complexes (granzyme B/perforin) engineered through polyphenol-membrane protein interactions. The platelet-based CDCs circulate systemically, selectively accumulating at surgical residual tumor sites. Subsequently, the accumulated platelets are activated by localized pathological signals. This activation triggers the inherent ability of platelets to release granules and microparticles. The therapeutic drug, associated with these platelet-derived microparticles, is then delivered to tumor cells, where it exerts its cytotoxic effects 121. The responsive strategies of CDCs are intrinsically endogenous and active, different from exogenous materials reacting to external signals. In recent years, drug-release strategies for BNDDSs have been continuously optimized, and cutting-edge studies have even achieved sub-organelle-level precision. Li et al. encapsulated a mitochondria-targeting triptolide palmitate (pTP) into ginseng-derived nanovesicles (G-NVs) and further conjugated melanoma-associated chondroitin sulfate proteoglycan-single-chain variable fragment (MCSP-scFv) onto their surface to construct G-NVs-MCSP/pTP, which exploits fatty-acid metabolic pathways to selectively transport the payload to tumor cell mitochondria and induce activation 122. These systems still fundamentally rely on static, preprogrammed chemical “triggers” (such as specific pH values or enzymatic cues) to initiate drug release. Once the carrier reaches any site that satisfies the predefined condition, release is automatically activated, lacking the capacity to dynamically sense and respond to the rapidly evolving intercellular interaction interfaces within the tumor microenvironment. Consequently, even the most advanced BNDDSs lack the capacity to dynamically sense and respond to the rapidly changing intercellular interaction landscapes that govern tumor progression and therapeutic response. CDCs intrinsically integrate drug release with living cellular behaviors, improving the level of spatiotemporal precision.

Cell-based intracellular drug delivery systems intrinsically couple drug release to cellular fate and endogenous activation pathways. For example, Ju et al. constructed a neutrophil-based delivery system by co-incubating neutrophils with Abraxane nanoparticles. The system relies on tumor inflammatory signals induced by radiotherapy to guide cellular homing; drug release then occurs after the cells have reached the lesion site via mechanisms such as cell apoptosis or post-activation content release. This demonstrates that the timing and extent of drug release depend on the endpoint of the natural physiological processes of cells, rather than being a pre-programmed, responsive event 123. CDCs conjugate drugs to the carrier cell surface via specialized linkers. These linkers are designed to respond to specific signals within the tumor microenvironment, thereby enabling controlled drug release while maximizing the retention of carrier cell viability and function. Noh et al. anchored gemcitabine onto the NK-cell membrane via a disulfide bond-containing linker. When these drug-conjugated NK cells migrate to tumor sites and recognize and attack cancer cells, the lysed tumor cells release high concentrations of glutathione (GSH). Acting as a “trigger signal,” the elevated GSH rapidly reduces and cleaves the disulfide linker, resulting in on-site, controllable release of gemcitabine from the NK-cell surface into the tumor microenvironment. Throughout this process, the intrinsic cytotoxicity of NK cells remains unaffected, and drug release is spatiotemporally synchronized with NK-cell-mediated tumor killing 124. Therefore, with superior control over drug delivery, predictable therapeutic response, and programmable system design, CDCs represent a more advanced paradigm than conventional intracellular drug-loading systems. Looking ahead, as our understanding of the tumor microenvironment's signaling networks deepens and as linker chemistry and cellular engineering continue to mature, CDCs hold considerable promise for broader application in precise tumor therapy.

## Applications of CDCs in Cancer Therapy

**Figure 7:**
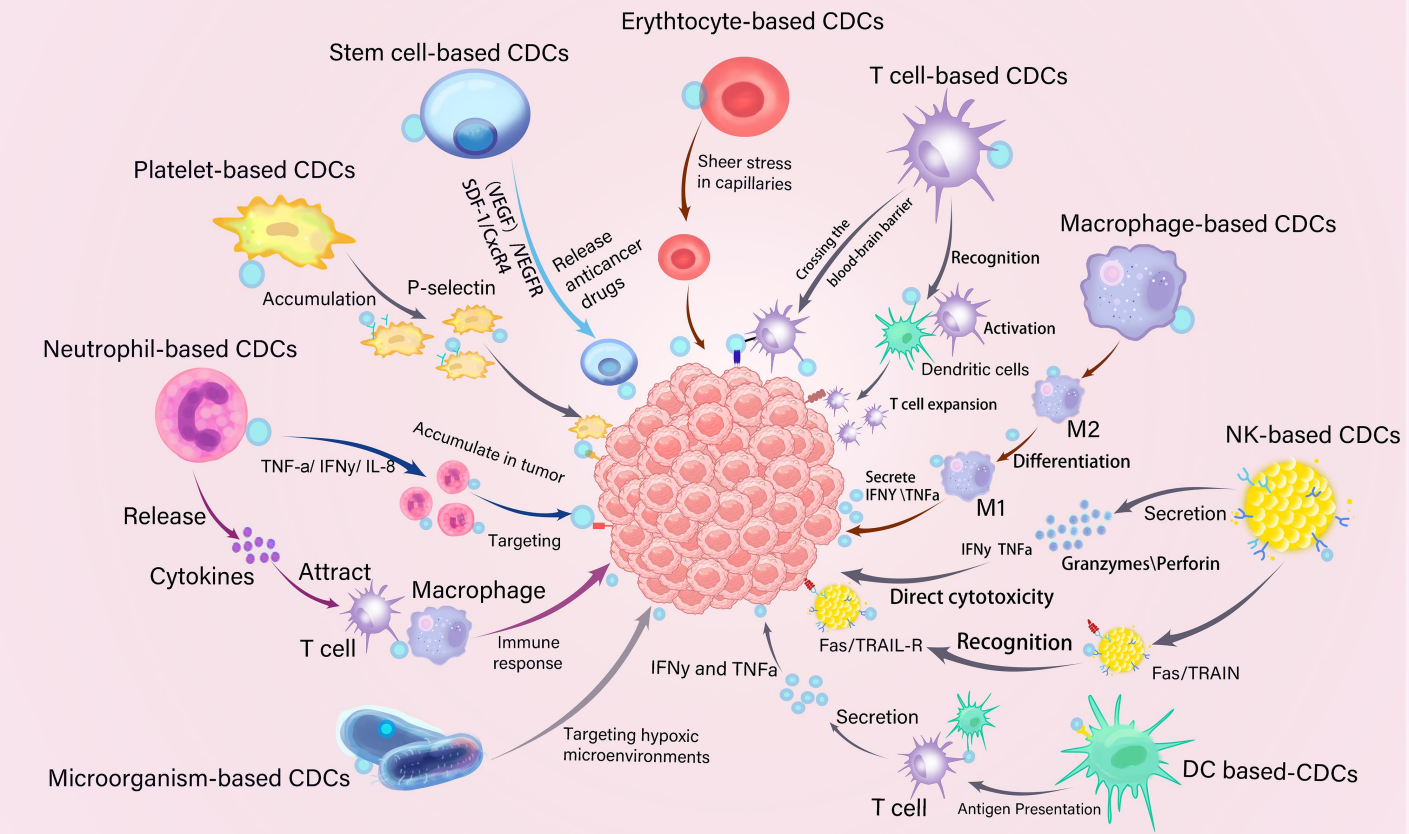
Schematics of CDCs used in cancer treatment, including erythrocyte-based CDCs, platelet-based CDCs, stem cell-based CDCs, neutrophil-based CDCs, macrophage-based CDCs, T cell-based CDCs, natural killer cell-based CDCs, dendritic cell (DC)-based CDCs, and microorganism-based CDCs. Fas, a type 1 transmembrane protein; IFNγ, interferon γ; TNF-α, tumor necrosis factor-alpha; IL-8, Interleukin-8; TRAIL, TNF-related apoptosis-inducing ligand; TRAILR, TRAIL receptor; SDF-1, stromal cell-derived factor-1; CXCR4, C-X-C chemokine receptor type 4. VEGF, vascular endothelial growth factor; VEGFR, VEGF receptor.

CDCs serve as versatile drug delivery systems for chemotherapy, immunotherapy, gene therapy, and combination therapy. Leveraging the innate biological advantages of living cells, CDCs have emerged as a transformative platform for cancer therapy **(Figure 7)**.

### Chemotherapy

Chemotherapy is one of the most widely used treatment methods for malignant tumors 125. The biggest disadvantage of chemotherapy drugs is that they attack cells indiscriminately and lead to characteristic side effects such as hair loss, vomiting, and bone marrow suppression. CDCs exploit the intrinsic homing capabilities of cells, enabling precise spatiotemporal drug delivery and significantly improving treatment efficacy. Takayama et al. successfully anchored doxorubicin-loaded liposomes (DOX-Lips) onto the surface of MSCs using the avidin-biotin complex method. The results demonstrated that MSC-DOX-Lips could efficiently transfer the surface-bound DOX-Lips to adjacent colon adenocarcinoma cells through direct cell-cell contact. Compared with equivalent doses of free doxorubicin or DOX-Lips alone, MSC-DOX-Lips exhibited significantly enhanced tumor-suppressive effects, indicating that CDCs can overcome the limitations of conventional chemotherapy in terms of tumor penetration and targeting efficiency 126. Kim *et al*. employed a chemical coupling method to combine DOX with carbon nanotubes (CNT) to form DOX-CNT complexes, which were then covalently conjugated to the surface of bone marrow-derived MSCs (BM-MSCs) to generate MSC-conjugates. *In vitro* co-culture experiments revealed that MSC-conjugate precisely releases DOX into lung cancer cells via intercellular contact and calcium signaling, leading to potent tumor cell elimination. *In vivo*, the MSC-conjugate group exhibited enhanced tumor regression and nearly doubled survival time compared to control groups. 127. These studies collectively highlight the ability of CDCs to exploit both tumor tropism and intercellular signaling of living cells for enhanced chemotherapeutic delivery within the TME.

Lymph node metastasis constitutes a major obstacle in solid tumor therapy 128. The unique vascular barrier of lymph nodes and their tumor-modified, immunosuppressive microenvironment result in subtherapeutic drug concentrations at sites of lymph node micrometastasis, thereby posing a significant challenge for chemotherapy efficacy 129. The strategy of CDCs addresses this by employing living cells with intrinsic lymph node homing capacity as delivery vehicles. T cells, particularly those engineered to express lymph node-homing receptors such as C-C chemokine receptor type 7 (CCR7), can actively enter lymph nodes via high endothelial venules 130. In a representative study, SN-38-loaded nano-capsules (NCs) were covalently attached to the plasma membrane of activated primary T cells. *In vivo*, the engineered T cells efficiently delivered SN-38 NCs to lymphoma-infiltrated tissues, including lymph nodes, spleen, and bone marrow. Compared with free-drug or NC-only administration, T cell-mediated delivery increased SN-38 concentration in lymph nodes by more than 90-fold and maintained these elevated levels for at least four days. Even at a low T cell-to-lymphoma ratio (1:20), SN-38 NC-loaded T cells effectively killed tumor cells without inducing self-cytotoxicity 109. The study clearly demonstrates that CDCs can utilize physiological lymphatic circulation and immune cell migration pathways to achieve precise targeting and controlled drug release towards lymph node metastases, thereby effectively overcoming the delivery barriers of chemotherapy drugs in the treatment of lymph node metastases. Beyond lymphatic dissemination, metastatic tumors frequently form disseminated lesions in distant organs, such as the lung, where heterogeneous architecture and stromal barriers further impede effective drug delivery and may contribute to therapeutic resistance. Li *et al*. exploited the property of macrophages targeting lung metastases and fabricated LD-MDS by coupling the legumain-specific propeptide of melittin (legM) and cytotoxic soravtansine (DM4) on the surface of macrophages 131. The system can specifically target metastatic lesions in lungs with controlled drug release for anti-metastasis therapy. Harnessing the highly expressed legumain in metastatic niches. LD-MDS can be actively recruited to lung metastasis sites and then responsively transform into DM4-loaded exosome-like nano-vesicles (DENs). Afterward, DM4 is released from the damaged 4T1 cancer cells to kill neighboring cancer cells, delivering anticancer drugs to additional cells. The experimental results showed that, at the same drug dosage, the therapeutic effect of LD-MDS was significantly superior to that of free chemotherapy drugs. LD-MDS achieved 86.7% inhibition rate of lung metastatic nodules, indicating that CDCs can significantly enhance the therapeutic effect of chemotherapy drugs in metastatic lesions.

The TME of pancreatic cancer is characterized by dense fibrosis, severe hypoxia, and immunosuppression, which obstructs the effective penetration and distribution of chemotherapeutic agents 132. Some natural and engineered bacteria possess the ability to selectively colonize and proliferate within hypoxic tumor regions. This inherent biological targeting property allows them to actively accumulate inside pancreatic cancer tissues. Felfoul *et al*. covalently conjugated SN-38-loaded lipid nanocarriers to magnetotactic bacterium *Magnetococcus marinus* strain MC-1. Guided by an externally applied magnetic field, these engineered bacteria can be precisely targeted to the hypoxic regions of the tumor. Their research demonstrated that magnetic guidance enables these bacterial vectors to effectively penetrate pancreatic tumor tissues 53. Beyond targeted accumulation, bacteria-based CDCs can also amplify intratumoral drug retention and distribution. Xie *et al*. conjugated DOX to the surface of* EcN* via an acid-sensitive linker. The experimental outcomes indicated that the drug accumulation of EcN-ca-DOX in tumor tissues (12.9% within 3 hours and 6.4% after 3 days) was higher than that of traditional nanocarriers. EcN-ca-DOX exhibited robust antitumor efficacy in both *in vitro* and *in vivo* models, effectively suppressing tumor growth, prolonging survival, and inducing apoptosis in cancer cells 133. By integrating biological targeting with physical guidance, responding to the tumor microenvironment for drug release, and achieving autonomous penetration and distribution, CDCs significantly enhance delivery efficiency and therapeutic efficacy in impermeable tumors, demonstrating clear translational potential in tumor chemotherapy.

Collectively, these studies demonstrate that CDCs represent a paradigm shift in chemotherapeutic delivery, transforming living cells from passive carriers into active, intelligent vectors capable of overcoming multiple biological barriers simultaneously. Importantly, CDC-mediated chemotherapy extends beyond simple enhancement of drug accumulation; it enables spatiotemporally controlled release, intercellular drug transfer, and microenvironment-responsive activation. This strategic shift enables CDC to optimized therapeutic potential of chemotherapy against primary tumors, lymphatic metastases, and even widespread distant metastases.

### Immunotherapy

#### Enhancing Effector Cell Persistence and Function

Adoptive T-cell transfer (ACT) is a breakthrough cancer immunotherapy. It involves expanding tumor-specific T cells outside the body and reinfusing them to directly kill tumor cells. However, its clinical application faces major hurdles. First, the immunosuppressive tumor microenvironment can cause the reinfused T cells to become exhausted and inactive, severely limiting their efficacy 134. Second, the systemic immunomodulatory drugs often used to enhance this therapy can improve T-cell function but frequently cause significant side effects due to their widespread action throughout the body 135. CDCs address these challenges by linking immunomodulatory drugs directly to the surface of therapeutic immune cells. This enables the simultaneous, targeted delivery of both the cells and the drug. In addition, the strategy achieves a dual functional and synergistic effect through this precise co-delivery. Specifically, the conjugated drug is passively concentrated at the tumor site with the effector cells. Concurrently, the drug actively "empowers" the carrier cells themselves by improving their metabolism and enhancing their proliferation potential, thereby delaying their exhaustion and optimizing their functional state. In a pivotal study, Avasimibe-loaded liposomes—designed to enhance T-cell membrane cholesterol metabolism—were conjugated to T cells via a combination of hydrophobic insertion and bioorthogonal click chemistry. In a GBM mouse model, Avasimibe-T-cell conjugates injected intravenously were able to target intracranial tumors and significantly inhibit their growth, with complete tumor elimination in 3/5 of the mice. This study demonstrated the dual advantages of CDCs in overcoming biological barriers and enhancing intrinsic cellular functions, providing a new strategy for the immunotherapy of solid tumors 136. Building on this foundation, researchers further explored the strategy of CDCs to precisely deliver immunostimulatory factors, aiming to sustain the expansion and maintain the effector functions of T cells. Stephan *et al*. engineered NPs encapsulating the cytokines IL-15 and IL-21, which were conjugated to Pmel-1 melanoma-specific T cells. Compared to T cells without nanoparticle modification, the conjugates exhibited significantly enhanced proliferation *in vivo* and achieved complete eradication of established B16F10 melanoma metastases in the lungs and bone marrow 137. Furthermore, to further enhance the precision and safety of treatment, the research focus has shifted from static, continuous cytokine stimulation toward dynamic, microenvironment-responsive smart release systems. For instance, IL-15SA nanogels can be anchored to T cells via a combination of electrostatic interactions and specific binding using an anti-CD45 antibody 138. The innovative core of this strategy is its “TCR signal-responsive” release mechanism—an intelligent design that enables on-demand delivery. Upon TCR-mediated recognition of a tumor cell, the high-concentration reducing microenvironment that forms within the immunological synapse triggers the release of IL-15SA from the nanogel. The design demonstrated significant antitumor efficacy and persistence in a melanoma model. Xie *et al*. developed an innovative approach to create nanogels by crosslinking IL-2/Fc protein via NHS-amine coupling chemistry. The nanogels covalently conjugate to T cell surfaces and specifically release IL-2/Fc in response to TCR activation. These nanogels enhanced T cell tumor infiltration and expansion capacity while promoting the generation of memory precursors and reducing T cell exhaustion 139. These studies indicate that future engineered interventions against T-cell exhaustion are moving toward dynamic, intelligent modulation. Linking cytokine delivery systems to the activation status of the T cells themselves can not only improve therapeutic efficacy and safety but also rebalance the immune response within the tumor microenvironment.

**Figure 8:**
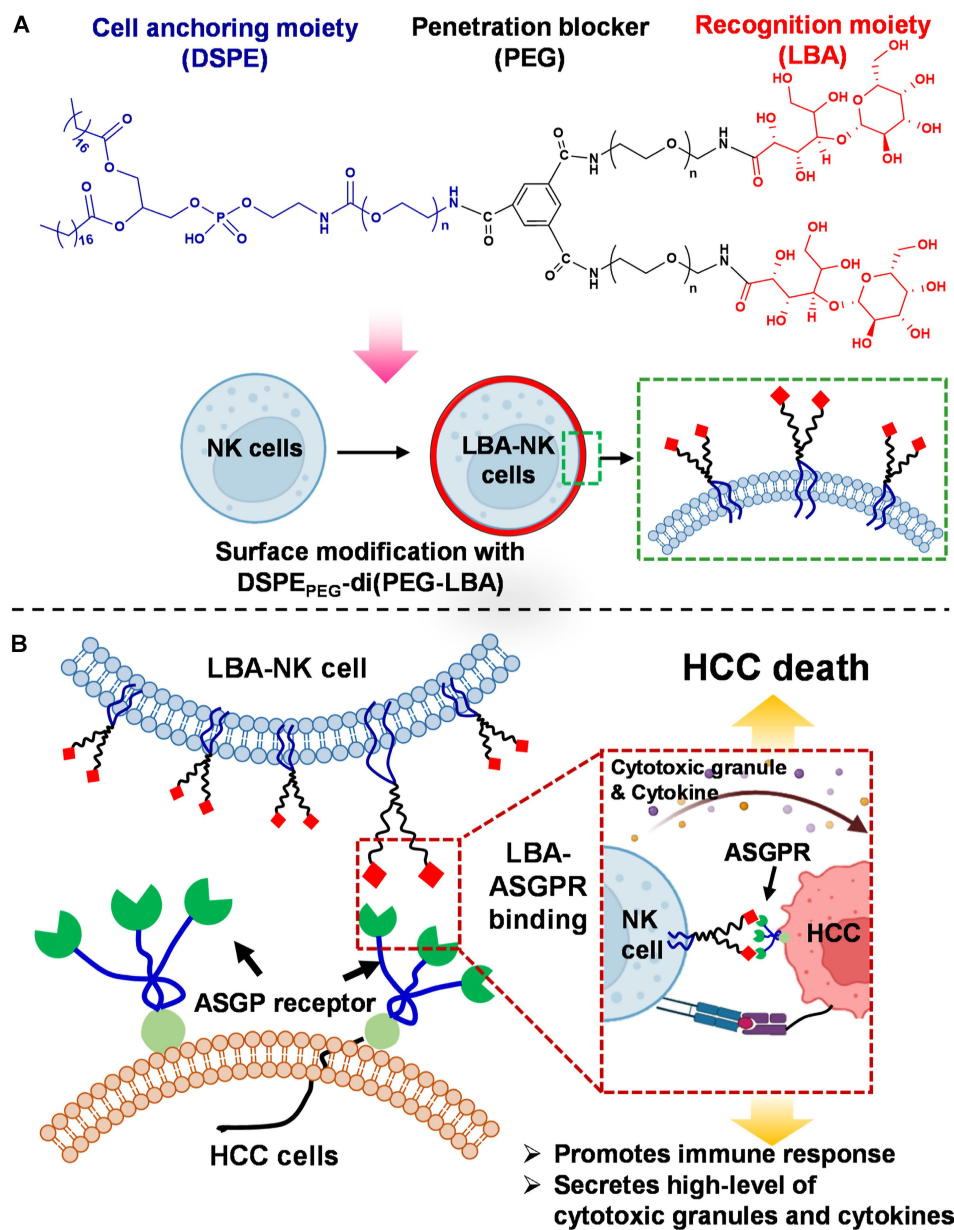
Schematic diagram illustration of NK cell surface engineering for target hepatocellular carcinoma. (A) Chemical structure of synthesized NK cell-coating biomaterial with representing lipid anchor, penetration blocker, and cancer recognition moiety and surface editing and presentation of LBA-mediated ligands onto NK cell membranes. (B) The mechanism of anticancer efficacy of LBA-NK cells via LBA-ASGPR binding. Adapted with permission from 141. Copyright 2023, American Chemical Society.

A key additional application of the CDCs is to endow innate immune cells, such as NK cells, with specific targeting capabilities. NK cells offer a distinct safety advantage because they do not cause graft-versus-host disease (GVHD) and carry a lower risk of cytokine release syndrome (CRS) 140. However, their ability to infiltrate and effectively target solid tumors remains limited. By conjugating tumor-specific targeting ligands to the surface of NK cells, this approach can transform them from “broad-spectrum killers” into “precision-guided effectors.” This retargeting strategy is particularly suited for tumor microenvironments, such as those in hepatocellular carcinoma, which are rich in immunosuppressive factors and contain clearly overexpressed target antigens. Jangid *et al*. designed a biomaterial-mediated *ex vivo* surface engineering technique for the membrane decoration of cancer recognition ligands onto NK cells. A lipid conjugate biomaterial they designed integrates three core functional units: a DSPE lipid segment for anchoring within the cell membrane, a PEG component to inhibit intracellular penetration, and a lactobionic acid (LBA) moiety that facilitates the identification of cancerous cells. The biomaterial was successfully applied to NK cell surfaces (LBA-NK). This modification endowed the NK cells with the ability to recognize the asialoglycoprotein receptor (ASGPR), which is abundantly expressed on the surface of hepatocellular carcinoma cells. Consequently, the LBA-NK exhibited significantly enhanced cytotoxicity against HepG2 cells **(Figure 8A-B)**. At an effector-to-target ratio of 10:1, the tumor cell lysis rate reached 56.3%, markedly higher than the 26.2% observed with unmodified NK cells. Furthermore, upon recognition of the target cells, the LBA-NK cells secreted elevated levels of perforin, granzyme B, and interferon-gamma (IFN-γ), indicative of potentiated immunologic activity 141.

The core strength of the strategy of CDCs lies in its ability to address two fundamental challenges simultaneously: the poor targeting of drugs and the functional limitations of therapeutic cells, thereby achieving a synergistic therapeutic effect. CDCs can be engineered for either “functional enhancement” or “targeted redirection,” making them adaptable to various immune cell types and clinical needs. Looking forward, with deepening insights into tumor immunobiology, the emergence of novel smart biomaterials, and the development of more efficient conjugation technologies, the CDC platform is poised to evolve. This evolution is expected to drive the clinical translation of next-generation cell immunotherapies that are safer, more precise, and more effective, offering new avenues for breakthroughs in treating solid tumors.

#### Targeted Delivery of Immune Checkpoint Inhibitors

Immune checkpoint inhibitors (ICIs), primarily targeting the programmed cell death protein 1 (PD-1)/PD-L1 axis, have revolutionized cancer therapy by reinvigorating antitumor immune responses across multiple malignancies 142. However, despite inducing durable clinical responses in a subset of patients, ICIs exhibit limited overall response rates and are frequently accompanied by immune-related adverse events arising from systemic immune activation, which collectively constrain their broader clinical application. CDCs exploit the intrinsic trafficking behavior, vascular interactions, and microenvironmental responsiveness of living cells, enabling spatially and temporally controlled delivery of immune checkpoint inhibitors. This unique capability is particularly advantageous for ICIs, whose therapeutic efficacy is highly context-dependent while systemic exposure often leads to immune-related adverse events (irAEs) 143. Platelet-based CDCs leverage the natural ability of platelets to accumulate at surgical wound sites, enabling the targeted local delivery of immune checkpoint inhibitors. Li *et al*. covalently conjugated anti-programmed cell death-ligand 1 (aPDL1) antibody with platelets to form P@aPDL1, which was then combined with Vadimezan, a vascular-disrupting agent. *In situ* activated platelets generate aPDL1-decorated platelet-derived microparticles (PMP) that diffuse within the tumor and elicit immune responses. The proposed combination increases 10-fold aPDL1 antibody accumulation in lung metastases as compared to the intravenous administration of the antibody and enhances the magnitude of immune responses, leading to improved antitumor effects 68. Beyond single-cell carrier systems, multistage living cells of CDCs have been explored to further refine tissue-specific immune checkpoint modulation. Hu *et al*. employed coupling technology to bind aPD-1 to platelets and subsequently attach them to the surface of hematopoietic stem cells via a click reaction 144. By leveraging the bone marrow-homing capability of HSCs and the site-specific activation of platelets, this platform achieved targeted delivery of anti-PD-1 to the bone marrow, enhancing the efficacy of immune checkpoint blockade. In murine leukemia models, the conjugate demonstrated potent antileukemic activity, extending survival and amplifying T cell-mediated immunity. The findings highlight that immune checkpoint inhibitors, in combination with multiple living cells, collectively constitute synergistic CDC platforms, overcoming the restricted tissue accessibility that limits conventional immune checkpoint therapies.

RBC-based CDCs, which lack major histocompatibility complex expression and exhibit exceptionally low immunogenicity alongside prolonged circulation lifespans, represent another promising platform of CDCs for immune checkpoint modulation. It has been demonstrated that PD-L1 molecules can be covalently conjugated onto the surface of RBCs through a one-step enzymatic ligation using recombinant asparaginyl ligase, which substantially reduces cellular damage while preserving immune tolerance. These PD-L1-decorated RBCs function as stable living cell-drug conjugates *in vivo* and provide a novel non-immune cell-based modality for modulating the PD-1/PD-L1 immune checkpoint axis in cancer immunotherapy 145. Although this strategy does not directly activate effector T cells, it provides a systemic, low-toxicity means of reshaping the dynamics of immune checkpoint signaling.

#### Remodeling the Immunosuppressive Microenvironment

The immunosuppressive TME constitutes a major obstacle to effective cancer immunotherapy and is characterized by the enrichment of M2-like tumor-associated macrophages (TAMs), high levels of inhibitory cytokines, and dysfunctional infiltration of effector T cells 146. CDCs have emerged as a powerful strategy to overcome these barriers by enabling the localized, sustained, and cell-mediated delivery of immunomodulatory agents directly within the TME, thereby actively reprogramming immunosuppressive niches. Unlike conventional immunomodulatory drugs, which often suffer from poor tumor accumulation, rapid systemic clearance, and off-target toxicity, CDCs leverage living immune or stromal cells as drug carriers, integrating the biological functions of the host cell with the therapeutic activity of the conjugated payload. Through this synergistic design, CDCs can selectively modulate the phenotypic and functional states of immune cells *in situ*, promoting macrophage repolarization, restoring effector T cell activity, and reshaping cytokine gradients within the tumor.

Shields et al. developed a particle system termed a “cell backpack” that stably attaches to the surface of macrophages and enables the sustained *in vivo* release of IFN-γ. Without compromising macrophage migratory capacity, this strategy locally drives macrophage polarization toward a pro-inflammatory M1 phenotype within the tumor microenvironment. Compared with free IFN-γ administration, anchoring immunostimulatory signals to living cells allows spatially confined cytokine release within tumors, thereby markedly enhancing the efficiency of local immune reprogramming. In a 4T1 breast cancer model, macrophages equipped with IFN-γ backpacks not only suppressed primary tumor growth but also significantly prolonged mouse survival, indicating that this system can indirectly reshape systemic antitumor immunity through modulation of innate immune cell states 104. CDCs can be further advanced into kinetically programmable immunostimulatory platforms, in which the dose, duration, and release profile of innate agonists are engineered at the cell surface to coordinate multicellular remodeling within the TME. Zhou et al. constructed Poly I: C-loaded PLP nanoparticles (PLP NPs) and conjugated them onto the surface of macrophages via maleimide-thiol coupling, generating PLP NP-decorated macrophages (MPLP). By controlling the number of PLP NPs attached to each macrophage, an initial “dose reservoir” of Poly I: C that could be delivered to tumor tissues was constructed. After accumulation within the tumor microenvironment, the PLGA-based nanocarriers exhibited pH-responsive sustained-release behavior in the acidic TME, with approximately 80% of the payload released over four days, enabling controlled local delivery of Poly I: C. The gradually released Poly I: C activated the carrier macrophages in an autocrine manner, driving their polarization toward an antitumor M1 phenotype. Subsequently, acting as paracrine signals, the sustained innate immune stimulation repolarized resident TAMs toward the M1 state and concurrently promoted dendritic cell maturation as well as the recruitment and activation of T cells and NK cells 63.

**Figure 9:**
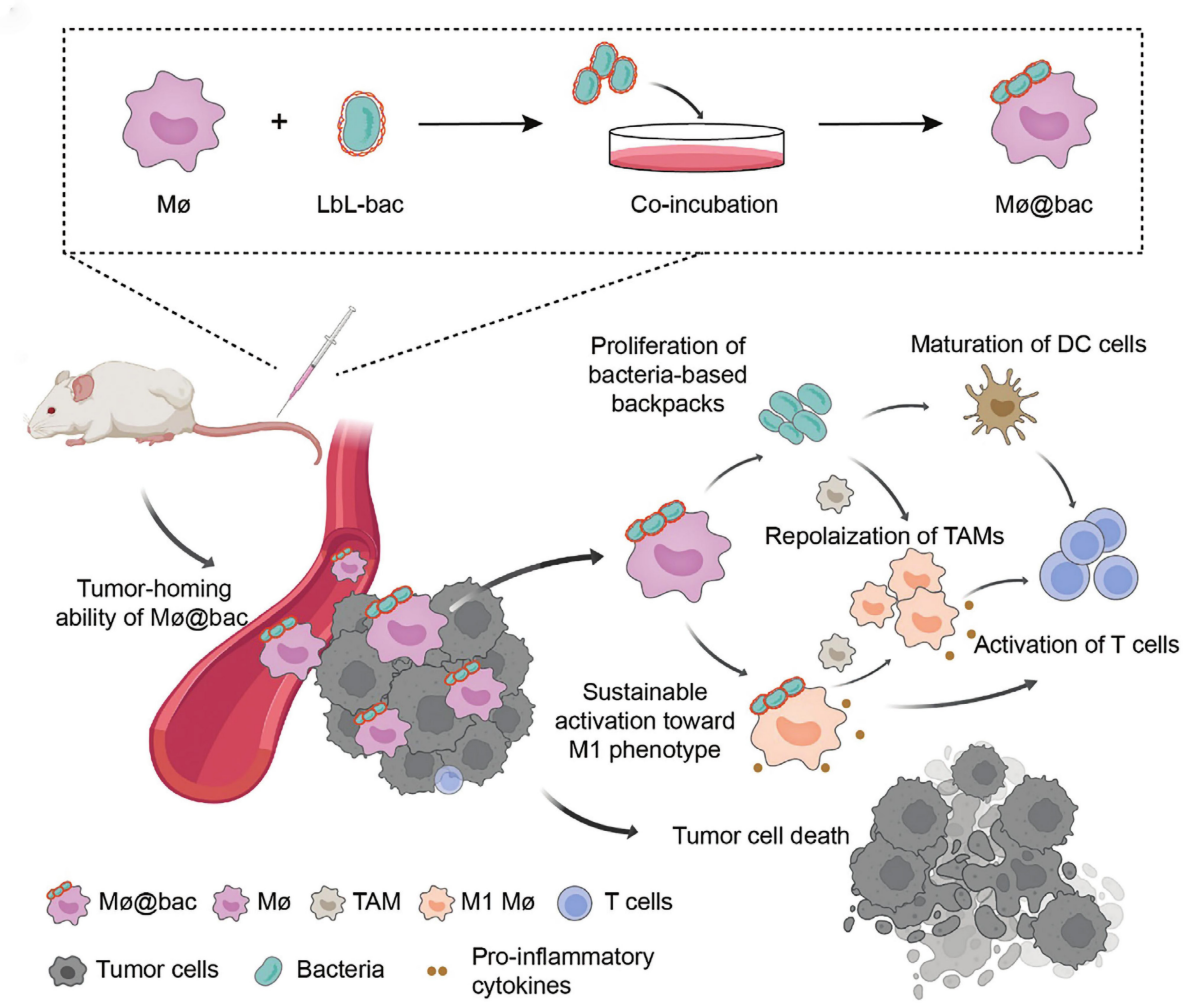
Preparation of Mø@bac and regulation of tumor immunosuppressive microenvironment mediated by Mø@bac. Adapted with permission from 147. Copyright 2023, Wiley-VCH.

In the latest study, CDCs utilized self-amplifying and immunogenic biological payloads conjugated to immune cells to achieve sustained remodeling of the immunosuppressive TME. For instance, An *et al*. developed a bacteria-based backpack (Mø@bac) that adheres to macrophages to enhance their functionality by leveraging the innate immunogenicity of bacteria **(Figure 9)** 147. These bacterial backpacks were enriched with pro-inflammatory factors and exhibited the ability to proliferate within tumor tissues, enabling sustained activation of macrophages toward the M1 phenotype. Moreover, Mø@bac demonstrated the ability to repolarize endogenous TAMs, remodeling the immunosuppressive TME. The sustained and localized release of pro-inflammatory factors from the backpacks creates a chemotactic gradient and a supportive niche that may enhance the recruitment and retention of both the engineered and endogenous macrophages within the tumor, amplifying the overall anti-tumor immune response. In subcutaneous and orthotopic 4T1 breast cancer murine models, Mø@bac exhibited significantly enhanced anti-tumor responses compared to macrophages alone or combined with free bacteria. Importantly, Mø@bac showed improved Biocompatibility with a reduced systemic inflammatory response. This innovative bacteria-based backpack strategy provides a novel pathway to modulate cellular phenotypes and alter the suppressive immune landscape, offering a powerful tool for CDCs.

Taken together, CDCs function as spatiotemporally programmable immune organizers that integrate intrinsic cellular behaviors with engineered payload presentation to reshape local immune ecosystems. Notably, the transition from cytokine anchoring to kinetically programmable nanoparticle depots and self-amplifying biological payloads underscores an inherent trade-off between increasing immunological potency and challenges in controllability, safety, and translational feasibility. Balancing immune amplification with regulatory precision will therefore be central to the rational design of next-generation CDCs for remodeling immunosuppressive TMEs.

### Gene Therapy

Traditional gene therapy for cancer is hindered by several challenges, including the instability of nucleic acids, poor target specificity, and low cellular uptake efficiency 148. Within the conceptual framework of CDCs, nucleic acids, viral vectors, and genetically programmable biological systems can all be regarded as functional “drugs”. CDCs, leveraging the innate homing ability, immune compatibility, and responsiveness of cellular carriers, present an innovative solution for overcoming these gene delivery hurdles. In the research conducted by Li *et al*., four conditionally replicating adenoviruses (CRAds) were constructed. The delivery of these CRAds was achieved by the fibers on the surface of adenoviruses recognizing and binding to specific receptors on the surface of DCs. After intravenous administration, dendritic cells carrying conditionally replicative adenoviruses (CRAds) home to tumor sites and release the viruses, thereby enabling localized, tumor-specific expression and amplification of therapeutic genes at the target lesions. Experiments showed that DCs coupling prostate cancer-specific CRAds (Ad-PPC-NCS, Ad-PPC-rmhTNF) exhibited more potent suppression on the viability of RM-1 cells than that of the prostate cancer-non-specific CRAds (Ad-PC, Ad-PC-rmhTNF). *In vivo*, intravenous injection of the prostate cancer-specific CRAd-loading DCs suppressed xenograft tumor growth, extended survival in tumor-bearing mice, and promoted T-cell activation 149. Gene therapy of CDCs extends beyond the delivery of nucleic acid drugs. It can also involve genetically engineering living cells to function as therapeutic carriers, empowering them to produce therapeutic agents *in situ*. Researchers have developed hypoxia-regulated early region 1A/B (E1A/B) gene constructs and adenoviruses whose replication is restricted by prostate-specific promoters, enabling selective proliferation in prostate tumor cells. Through genetic engineering techniques, macrophages were co-transfected with adenoviruses and hypoxia-response element (HRE)-E1A/B plasmids. This allowed macrophages to transport and express adenoviruses, which then replicated actively under hypoxic conditions, leading to targeted adenoviral amplification within tumor cells and efficient tumor lysis 150. Taken together, CDC-mediated gene therapy is not simply a better delivery strategy, but a transition toward programmable living therapeutics.

### Combination Therapy

**Figure 10:**
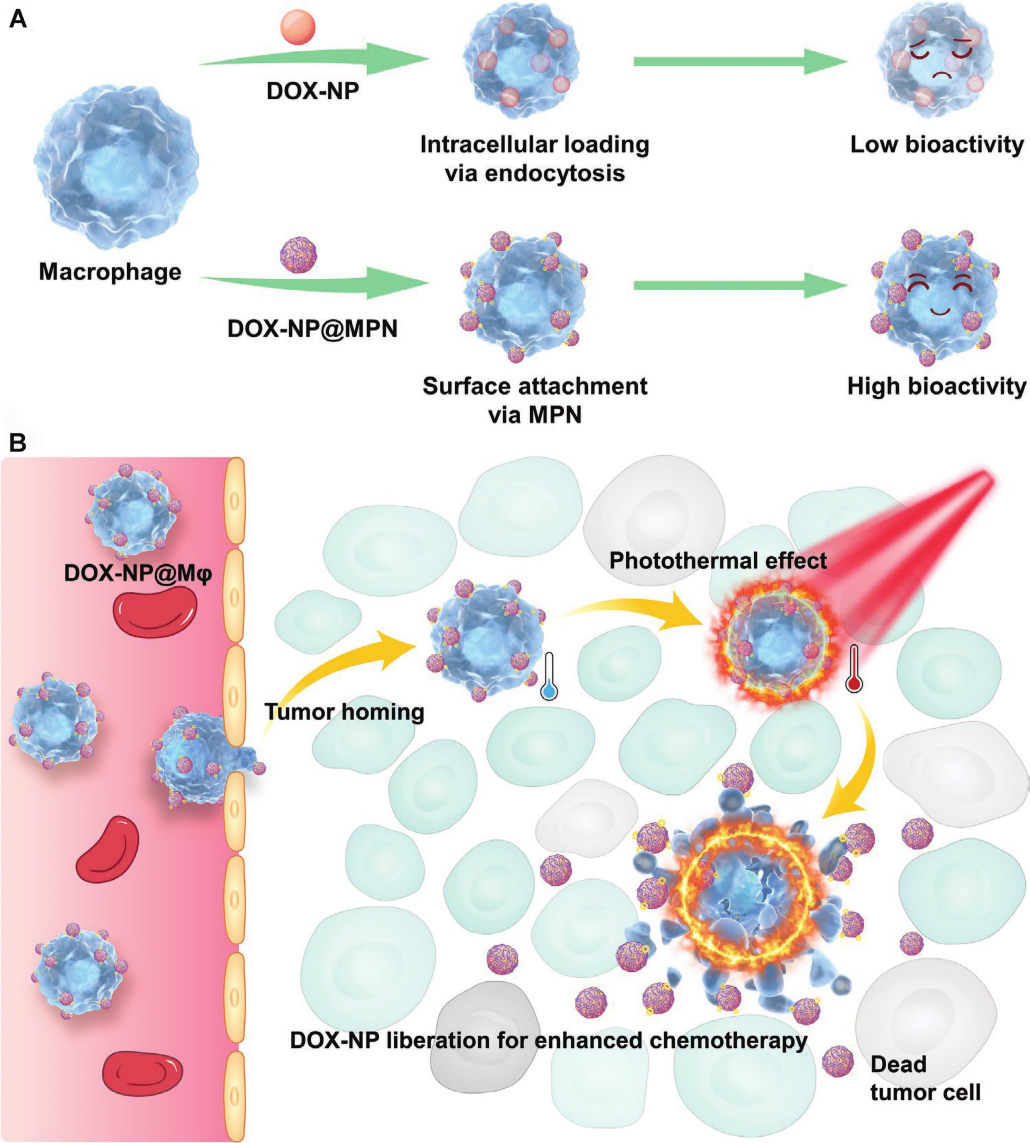
(A) Schematic illustration of the construction of macrophages with surface-attached DOX-NP via MPN. High bioactivities are maintained compared to the conventional intracellular loading via endocytosis. (B) The therapeutic performance of DOX-NP@Mϕ in tumors. Adapted with permission from 151. Copyright 2023, Wiley-VCH GmbH.

Triggering mechanisms based on endogenous cellular behavior highlight the substantial potential of CDCs in precision therapy. Beyond relying solely on endogenous cellular responses, CDC-based combination therapies increasingly incorporate externally applied physical stimuli as triggers to coordinate drug release and therapeutic mechanism activation with high spatiotemporal precision. A research team innovatively utilized the coordination interaction between divalent metal ions (such as Cu²⁺, Mn²⁺, Co²⁺, Zn²⁺, etc.) and polyphenols (tannic acid) to construct an Mϕ- delivery system with DOX-loaded nanoparticles on the macrophage surface (DOX-NP@Mϕ)** (Figure 10A-B)**. Importantly, the metal-phenolic network endowed the surface-bound nanoparticles with pronounced photothermal conversion capability. Upon NIR laser irradiation at the tumor site, localized photothermal heating induced structural disruption of the macrophage membrane and weakened the coordination interactions anchoring the nanoparticles, thereby triggering rapid detachment and release of DOX-loaded nanoparticles into the tumor microenvironment. The liberated nanoparticles were subsequently internalized by tumor cells at markedly enhanced levels, resulting in significantly increased intratumoral drug accumulation. *In vivo* studies demonstrated that this photothermally triggered, on-demand nanoparticle release led to pronounced suppression of breast tumor growth and prolonged survival in murine models, outperforming non-irradiated controls 151. The work represents a strategy of CDCs in which chemotherapy is synergistically combined with photothermally programmable release, enabling spatially confined, temporally precise drug activation while maintaining carrier cell functionality.

In recent years, CDC-mediated combination therapies have been further advanced by integrating physical triggers, programmed cell death induction, and immune modulation strategies, thereby amplifying the overall antitumor efficacy of combinatorial treatments. Ding *et al*. developed semiconducting polymer nano-therapeutics (SPCFe/siP) that can achieve efficient delivery into orthotopic glioma sites via neutrophil-mediated BBB transport for sono-activatable ferroptosis-immunotherapy. Such SPCFe/siP contains a semiconducting polymer, PD-L1 siRNA, and Fe₃O₄ NPs loaded into a singlet oxygen (¹O₂)-cleavable nanocarrier with surface conjugation of a neutrophil targeting ligand. Upon intravenous injection, the NEs mediated effective delivery across the BBB to the GBM. Ultrasound activation then triggered the release of singlet oxygen (¹O₂) from the NPs, inducing both ferroptosis and immunogenic cell death. Concurrently, the released PD-L1 siRNA downregulated PD-L1 expression, thereby enhancing the antitumor immune response. This combined action resulted in potent suppression of glioblastoma growth and a significant extension of survival in mouse models 49. The study integrates ferroptosis and immune checkpoint blockade through ultrasound-triggered activation, highlighting the ability of CDCs to synchronize multimodal therapy with deep-tissue accessibility and immune modulation.

In conclusion, a summary of CDCs for cancer therapy is provided in **Table 2**, with illustrative examples for each of the aforementioned therapeutic interventions.

## Clinical Trials of CDCs in Cancer Therapy

This section reviews ongoing clinical trials, demonstrating the promise of CDCs in cancer treatments and emphasizing the need for robust strategies to bridge the gap between research and patient care. Several clinical trials are underway to evaluate the safety, pharmacokinetics, and therapeutic efficacy of CDCs in cancer treatment. As of 2025, CDCs have entered clinical evaluation, primarily leveraging autologous cell carriers such as erythrocytes, T cells, DCs, and platelets (**Table 3**). An RBC-conjugated therapy combining 4-1BB ligand, IL-12, and human papillomavirus type 16 antigens (HPV-16 antigens) for cervical and head/neck cancers (NCT04672980). Early results showed favorable tolerability but limited efficacy, leading to trial termination. Similarly, RBCs coupled with 4-1BB ligand and IL-12 were tested for advanced solid tumors (NCT05219578), but the trial was discontinued due to insufficient tumor regression. This limited efficacy is hypothesized to stem from the dense stromal tissue and aberrant vasculature of solid tumors, which severely impede red blood cell extravasation and tissue penetration. Consequently, the local intratumoral concentration of the therapeutic agents likely failed to reach the necessary therapeutic threshold 157. Furthermore**,** erythrocytes were conjugated with αPD-1 (NCT06528249) and PD-1/PD-L1 inhibitor (PDx) (NCT05707325). These combinations are recruiting or not yet recruiting, evaluating the efficacy of erythrocyte-αPD-1/PDx conjugates in patients with advanced malignancies. IL-15-conjugated T cells (NCT03815682) demonstrated enhanced tumor infiltration but faced challenges in CRS, leading to early termination. The strategies are being explored to address this limitation. First, incorporating genetically engineered safety mechanisms, such as inducible “suicide switches” expressed on CAR-T cells, can allow for the controlled elimination of overactive cell populations upon the onset of CRS 158. Second, combination therapies can be employed to overcome the immunosuppressive TME. For instance, pairing cellular therapies with small-molecule inhibitors targeting pathways like adenosine or TGF-β signaling can help remodel the local immune landscape to support therapeutic efficacy 159,160. CAR-T cells conjugated with PD-1 inhibitors (NCT02546167) showed promise in hematologic malignancies, with the study successfully completed. DCs conjugated with recombinant human fusion proteins and oxidized polymannos (HFP-OP) induced antigen-specific T-cell responses in ovarian cancer (NCT01617629). Preliminary results suggest promising safety and efficacy in epithelial ovarian cancer. Clinical trials involving dendritic cell vaccine (Cvac) demonstrated favorable safety and immunogenicity, with some patients exhibiting significant T-cell IFN-γ responses and delayed-type hypersensitivity reactions, suggesting robust immune activation. The clinical study of NCT01521143 has been completed. However, subsequent Phase II trials (NCT02310971) were terminated, reflecting the difficulty of reproducing preclinical efficacy in larger cohorts. Moreover, a T cell membrane-anchored tumor-targeted IL12 -modified TIL cell therapy (attIL12-TIL) trial, evaluating the anti-tumor efficacy achieved following adoptive transfer of T cell membrane- anchored tumor- targeted IL12 (attIL12)-TIL cell therapy in combination with cyclophosphamide in participants with advanced/metastatic soft tissue or bone sarcomas advanced malignancies, has been registered but is not yet recruiting (NCT06474676). It reflects the ongoing interest in CDC-based approaches. A phase I clinical trial of an Infusion of autologous T cells genetically engineered with a chimeric receptor to target the follicle-stimulating hormone receptor in patients with recurrent ovarian cancer (NCT05316129). The purpose of this study is to evaluate the safety of treatment with autologous T cells genetically modified to express a chimeric endocrine receptor (CER) targeting the follicle-stimulating hormone receptor (FSHR). Participants will receive escalating doses of FSHCER T cells to determine the maximum tolerated dose (MTD). The clinical study has been recruiting. The exploration of CDC-based immunotherapies continues to expand. Platelet-conjugated IL-2 (NCT05829057) is currently recruiting patients with advanced malignancies, aiming to exploit the intrinsic wound-homing properties of platelets to deliver IL-2 post-surgically, thereby preventing tumor recurrence. Currently, the majority of clinical trials concerning CDCs remain in Phase I or II, characterized by restricted sample numbers and inadequate long-term follow-up data. The longevity of their therapeutic benefits, safety profiles, and possible immune-related side events necessitates more research.

## Challenges and Perspectives

CDCs are a novel delivery platform that combines the biological intelligence of living cells with the therapeutic power of drugs, offering a promising path for cancer therapy. However, translating CDCs from basic research to widespread clinical use remains challenging. As a unique type of “living” drug, the safety and controllability of cell production are the most critical, yet vulnerable, part of the entire technology chain of CDCs. The primary challenge stems from the inherent heterogeneity of cell sources and the difficulty in standardizing production processes. Cell sources can generally be classified into three main categories: autologous, allogeneic, and xenogeneic 161. Autologous cells offer superior biocompatibility and minimal immunogenicity, yet their clinical utility is constrained by patient-dependent variability 162. Xenogeneic cells, however, may trigger host immune rejection, making them less than desirable candidates. Allogeneic cells may serve as a promising alternative for the development of CDCs. However, immune rejection of allogeneic cells has been a long-standing challenge that necessitates the use of immunosuppressive agents in the clinic 163. To mitigate immune rejection and enhance cell survival, biomaterial-based encapsulation strategies have been explored. For example, the encapsulation of individual MSCs within ultrathin alginate microgels, which bolsters both survival and functionality 164,165. Further enhancements in cell viability have been achieved by embedding hydrogels with critical bioactive factors, such as cytokines, adhesion ligands, and extracellular matrix proteins. Studies demonstrate that collagen-based scaffolds markedly improve the persistence of transplanted cardioblasts by facilitating vascular infiltration into the porous structure 166. Similarly, coating MSCs with silica layers has been shown to augment their survival in suspension cultures by providing a favorable substrate. Beyond physical scaffolds, biomaterials functionalized with pro-survival peptides, as in the attachment of pro-survival peptides to a collagen matrix to enhance stem cell viability post-transplantation 167.

The expansion and activation of cells represent critical stages in the manufacturing process. Many immune cells, such as naïve T cells or resting macrophages, must undergo a complex* ex vivo* protocol to achieve the therapeutic dose required for treatment. This step, however, is highly susceptible to batch-to-batch variability. Key cellular characteristics, including activation status, proliferative capacity, and final phenotype, can be subtly altered by variations in culture conditions, cytokine formulations, and technician technique. Consequently, the development of closed, automated bioreactor systems that precisely regulate temperature, pH, nutrient supply, and metabolic waste is essential for scalable and consistent industrial production. Significantly, the integration of artificial intelligence (AI) is instrumental in accelerating the research, development, and manufacturing of CDCs. Machine learning involves autonomously extracting high-dimensional features from large-scale, complex datasets. This capability is particularly valuable for modeling intricate bioprocesses, such as cell culture dynamics and drug delivery mechanisms 168. For instance, ensemble learning and collaborative filtering techniques can significantly enhance the screening of optimal drug conjugate candidates, thereby expediting the optimization of personalized treatment regimens 169,170. QUANIC uses large-scale, multimodal clinical data to develop models predicting response and resistance to Immunomodulators promoting the development of CDCs in personalized cancer immunotherapy 171. In addition, deep learning-based methods can be applied to optimize drug delivery systems and dynamically adjust manufacturing processes. These intelligent approaches are anticipated to enhance the preparation efficiency and predictive accuracy of CDCs, while also enabling real-time optimization and dynamic regulation of the manufacturing process, improving the overall consistency, controllability, and clinical applicability of CDCs production.

Developing efficient methods of conjugation is crucial for achieving targeted drug delivery. Recent advances in micro- and nanotechnologies offer promising solutions to improve conjugation efficiency while enhancing process controllability. Notably, a sonodynamic strategy employing bulk acoustic wave (BAW) resonators integrated within microfluidic chips has enabled the *in situ* cong drug-loaded NPs onto the surface of RBCs in a single-step process 172. By harnessing acoustic streaming effects, this platform precisely regulates the interactions between cells and nanoparticles while simultaneously removing excess particles through designated waste outlets, eliminating the need for repetitive washing or centrifugation. Beyond its technical novelty, this acoustofluidic approach exemplifies a broader shift toward closed, automated, and inline manufacturing platforms that may be pivotal for the standardized and scalable production of CDCs.

The *in vivo* behavior of CDCs remains a critical and unresolved challenge. Upon systemic administration, interactions with blood components may lead to the formation of a protein corona on the cell surface, thereby altering its physicochemical properties, masking targeting ligands, and modulating immune recognition. Such dynamic surface remodeling can compromise targeting specificity and delivery efficiency, and may even introduce unanticipated safety risks. Therefore, future design of CDCs should move beyond static *in vitro* optimization and incorporate systematic evaluation of *in vivo* surface remodeling, biodistribution, and immune interactions as integral components of the design process. Developing predictive models that link in vitro properties to *in vivo* behavior will be crucial for improving the reliability and safety of CDC-based therapeutics.

To that end, extensive interdisciplinary collaborations integrating knowledge and technologies from a variety of fields, including materials chemistry, biomedicine, and computer science, are necessary to realize the further development of CDCs for research and applications in cancer therapy.

## Summary

Although strategies such as BNDDS have partially improved the pharmacokinetics of drugs, they remain structurally constrained by inherent limitations. These include interfacial instability between the membrane and core, rapid clearance by the RES, inadequate penetration into solid tumors, and the significant gap between preclinical results and clinical translation. CDCs combine living cells with therapeutics, leveraging the biological properties of cells to achieve precise targeting, which circumvents the structural bottlenecks associated with BNDDS. This review examines the construction of CDCs as drug delivery systems, including carrier cells, coupling components, and conjugation strategies*.* Additionally, it emphasizes the advantages of CDCs as drug delivery systems. Furthermore, we illustrate the important role of CDCs in modern cancer therapies, such as chemotherapy, immunotherapy, gene therapy, and combination therapy. CDCs leverage live cells as carriers to enable the targeted, high-precision delivery of chemotherapeutic and immunomodulatory agents, as well as tissue-specific gene transfer and expression. By harnessing the synergy between the cellular carrier and its therapeutic payload, CDCs can amplify immune responses. Furthermore, integrating the inherent targeting capabilities of living cells with exogenous physical stimuli permits the creation of a spatiotemporally controlled drug release platform. Translating CDCs from basic research to widespread clinical use remains challenging. The foremost challenge lies in the standardized manufacturing and quality control of CDCs. Future progress will critically depend on the interdisciplinary integration of artificial intelligence, automation technologies, and smart biomaterials to overcome production bottlenecks and achieve precise, controllable manufacturing processes.

## Figures and Tables

**Table 1:** Comparative evaluation of cellular for CDCs.

Carrier type	Representative advantages	Key limitations	Preferred applications and relative positioning
Red blood cells	Long circulation half-life; excellent biocompatibility; large surface area for drug conjugation	Lack of active tumor homing; limited penetration into solid tumors	Well-suited for long-circulating delivery systems and sustained drug release, with high safety but limited targeting precision
Platelets	Natural accumulation at inflammatory and post-surgical tumor sites; effective delivery of immunomodulators	Short lifespan; potential risk of thrombosis and tumor-platelet interactions	Suitable for postoperative tumor recurrence prevention and localized immunotherapy
Mesenchymal stem cells	Strong tumor tropism; potent paracrine activity; low immunogenicity	Potential tumor-promoting effects; prolonged retention *in vivo*	Favorable for targeted delivery to dense or stromal-rich tumors, but safety requires careful control
T cells	Antigen-specific tumor recognition; potent cytotoxicity; immune memory formation	Risk of cytokine release syndrome; functional exhaustion	Highly effective for precision-targeted and immune-amplifying, with safety as a major concern
Natural killer cells	Antigen-independent tumor killing; rapid immune response; reduced cytokine storm risk	Limited *in vivo* persistence; scalability challenges	Suitable for broad-spectrum antitumor CDCs with improved safety compared to T cells
Macrophages	Strong phagocytic activity; infiltration into hypoxic tumor regions	Susceptible to immunosuppressive polarization in TME	Advantageous for deep tumor penetration and microenvironment-responsive delivery
Neutrophils	Rapid chemotaxis toward tumors and inflammatory signals; ability to cross biological barriers	Extremely short lifespan; phenotypic instability	Effective for rapid, inflammation-driven delivery, but limited by poor controllability
Dendritic cells	Superior antigen presentation; robust activation of adaptive immunity	Limited direct cytotoxicity	Primarily used for immunostimulatory CDCs with vaccine-like functions
Microorganisms	Active motility; preferential colonization of hypoxic and necrotic tumor regions; genetic programmability	High immunogenicity; biosafety concerns; risk of systemic infection	Attractive for deep tumor penetration and hypoxia-targeted therapy, but clinical translation requires strict safety control

**Table 2:** Summary of representative CDCs for the treatment of cancers.

Types of CDCs	Engineered methods	Conjugated compositions	Therapeutic Modalities	Indications	Refs
Red blood cell-based CDCs	Physical adsorption	DOX-loaded PLGA NPs	Chemotherapy	Lung metastases	152
Platelet-based CDCs	Covalent bonding	aPD-1	Immunotherapy	Leukemia	68
	Covalent bonding	aPDL1	Immunotherapy	Breast cancer Lung metastases	143
	Ligand-receptor binding	DTX-loaded glycopolymeric micelles	Chemotherapy	Breast cancer	21
Mesenchymal stem cell- based CDCs	Antibody-antigen binding	Anti-CD90-CNT-DOX	Chemotherapy	Lung cancer	127
	Biotin-streptavidin	DOX-Lips	Chemotherapy	Colon adenocarcinoma	126
Neutrophil-based CDCs	Receptor-ligand binding	PIX -PSL	Immunotherapy	Lung cancer	59
	Receptor-ligand binding	SPCFe/siP	Combination therapy	Orthotopic glioma	49
T cell-based CDCs	Covalent bonding	SN-38-loaded nanocapsules	Chemotherapy	Lymphoma	109
	Maleimide-thiol coupling reaction	Multilamellar lipid NPs with cytokines IL-15SA and IL-21	Immunotherapy	Pulmonary and bone marrow metastasis of melanoma	137
	Covalent bonding	Redox-responsive IL-2/Fc nanogel	Immunotherapy	Pulmonary metastasis of melanoma	153
	Hydrophobic chain insertion and tetrazine-BCN click reaction	Avasimibe-loaded liposome	Immunotherapy	Melanoma Glioblastoma	136
Macrophage-based CDCs	Peptide-receptor binding	Peptide liposomes co-loaded with DOX and sorafenib	Chemotherapy	Breast cancer	154
	Genetic engineering	HRE-E1A/B Plasmid CMV-AdV5-GFP	Gene therapy	Prostate cancer	150
	Electrostatic interactions Receptor-ligand binding Physical adsorption	Cell backpack containing IFN-γ	Immunotherapy	Breast cancer	104
	Metal ion-ligand binding	DOX-NP@MPN	Combination therapy	Breast cancer	151
	Maleimide-thiol coupling reaction	Poly(I:C) loaded with PLP NPs	Immunotherapy	Triple negative breast cancer	63
	Covalent bonding	DMPE-PEG-S-S-DM4 DMPE-PEG-legM	Chemotherapy	Breast cancer lung metastases	131
Dendritic cell-based CDCs	Receptor-ligand binding	Replicating adenoviruses	Gene therapy	Prostate cancer	149
	Maleimide-thiol covalent bond	cMLVs	Chemotherapy	Ovarian cancer	155
Bacteria-based CDCs	Covalent bonding	DOX	Chemotherapy	Breast cancer	133
	Biotin-streptavidin	DTX-loaded HA-PE microbeads	Chemotherapy	Breast cancer	156
	Covalent bonding	SN-38-loaded nanoliposomes	Chemotherapy	Colorectal Cancer	53

DOX, doxorubicin; PLGA NPs, poly (lactic-co-glycolic acid) nanoparticles; aPD-1, anti-programmed cell death protein 1 antibody; aPDL1, anti-programmed cell death protein ligand 1 antibody; DTX, docetaxel; CNT, carbon nanotube; Lips, liposomes; PIX, paclitaxel; PSL, phosphatidylserine liposome; SPCFe, superparamagnetic iron oxide nanoparticles; siP, siRNA targeting PD-L1. IL-15SA, interleukin-15 superagonist; IL-21, interleukin-21; IL-2/Fc, interleukin-2/fusion protein; BCN, bicyclo[6.1.0]nonyne; HRE, hypoxia-response element; CMV, cytomegalovirus (promoter); AdV5, Adenovirus serotype 5; GFP, green fluorescent protein; IFN-γ, interferon-gamma. MPN, metal-phenolic network; Poly(I: C), polyinosinic: polycytidylic acid; PLP NPs, poly(lactic acid)-poly(ethylene glycol) nanoparticles; DMPE-PEG: 1,2-dimyristoyl-sn-glycero-3-phosphoethanolamine-N-[methoxy(polyethylene glycol)]; DM4: cytotoxic soravtansine; legM, legumain-specific propeptide of melittin; HA-PE, hyaluronic acid-phosphatidylethanolamine. SN-38,7-ethyl-10-hydroxycamptothecin.

**Table 3:** Clinical study of CDCs in cancer.

Conditions	Conjugated compositions	Carrier source	NCT number	Phase	Clinical status
Hematologic malignancy	PDx	Erythrocytes	NCT05707325	Phase 1	Recruiting
Hematologic malignancy	αPD-1	Erythrocytes	NCT06528249	Phase 1	Not yet recruiting
Cervical cancer Head neck cancer Anal cancer	4-1BB ligand IL-12, HPV-16 antigen	Erythrocytes	NCT04672980	Phase 1	Terminated
Advanced solid tumors	4-1BB ligand, IL-12	Erythrocytes	NCT05219578	Phase I-II	Terminated
Multiple myeloma	4-1BB ligand	T cells	NCT02546167	Phase 1	Completed
Bone sarcoma	IL12	T cells	NCT06474676	Phase 1	Not yet recruiting
Ovarian cancer	NO	T cells	NCT05316129	Phase 1	Recruiting
Lymphoma Solid Tumor	IL-15	T cells	NCT03815682	Phase 1	Terminated
Epithelial ovarian cancer	HFP-OP	Dendritic Cells	NCT01617629	Phase 2	Completed
Pancreatic Carcinoma	HFP-OP	Dendritic Cells	NCT02310971	Phase 2	Withdrawn
Esophagus cancer Bladder cancer Liver cancer Ovarian cancer	IL-2	Platelets	NCT05829057	Phase 1	Recruiting

PDx, anti-programmed cell death protein 1 antibody and programmed cell death protein 1 / programmed cell death protein ligand1 inhibitor; αPD-1, anti-programmed cell death protein 1 antibody; IL-12 / IL12, interleukin-12; HPV-16, human papillomavirus type 16; IL-15, interleukin-15; HFP-OP, human fusion proteins and oxidized polymannose; IL-2, interleukin-2.
